# Characterizing Antimicrobial Resistant *Escherichia coli* and Associated Risk Factors in a Cross-Sectional Study of Pig Farms in Great Britain

**DOI:** 10.3389/fmicb.2020.00861

**Published:** 2020-05-25

**Authors:** Manal AbuOun, Heather M. O’Connor, Emma J. Stubberfield, Javier Nunez-Garcia, Ellie Sayers, Derick W. Crook, Richard P. Smith, Muna F. Anjum

**Affiliations:** ^1^Department of Bacteriology, Animal and Plant Health Agency, Weybridge, United Kingdom; ^2^National Institute for Health Research, Health Protection Research Unit, University of Oxford in Partnership with Public Health England (PHE), Oxford, United Kingdom; ^3^Department of Epidemiological Sciences, Animal and Plant Health Agency, Weybridge, United Kingdom; ^4^Modernising Medical Microbiology Consortium, Nuffield Department of Medicine, John Radcliffe Hospital, University of Oxford, Oxford, United Kingdom

**Keywords:** antimicrobial resistance, multidrug resistance, plasmids, epidemiology, risk factor analysis, pig farms, *Escherichia coli*, Great Britain

## Abstract

Combatting antimicrobial resistant (AMR) using a One-Health approach is essential as various bacteria, including *Escherichia coli*, a common bacteria, are becoming increasingly resistant and livestock may be a reservoir. The AMR gene content of 492 *E. coli*, isolated from 56 pig farms across Great Britain in 2014–2015, and purified on antibiotic selective and non-selective plates, was determined using whole genome sequencing (WGS). The *E. coli* were phylogenetically diverse harboring a variety of AMR profiles with widespread resistance to “old” antibiotics; isolates harbored up to seven plasmid Inc-types. None showed concurrent resistance to third-generation cephalosporins, fluoroquinolones and clinically relevant aminoglycosides, although ∼3% harbored AMR genes to both the former two. Transferable resistance to carbapenem and colistin were absent, and six of 117 *E. coli* STs belonged to major types associated with human disease. Prevalence of genotypically MDR *E. coli*, gathered from non-selective media was 35% and that of extended-spectrum-beta-lactamase *E. coli* was low (∼2% from non-selective). Approximately 72.6% of *E. coli* from ciprofloxacin plates and only 8.5% from the other plates harbored fluoroquinolone resistance due to topoisomerase mutations; the majority were MDR. In fact, multivariable analysis confirmed *E. coli* purified from CIP enrichment plates were more likely to be MDR, and suggested MDR isolates were also more probable from farms with high antibiotic usage, specialist finisher farms, and farms emptying their manure pits only after each batch. Additionally, farms from the South East were more likely to have MDR *E. coli*, whereas farms in Yorkshire and the Humber were less likely. Future investigations will determine whether suggested improvements such as better biosecurity or lower antimicrobial use decreases MDR *E. coli* on pig farms. Although this study focuses on pig farms, we believe the methodology and findings can be applied more widely to help livestock farmers in the United Kingdom and elsewhere to tackle AMR.

## Introduction

*Escherichia coli* is a key species associated with AMR, including multidrug resistance (resistance to 3 or more antimicrobial classes; MDR) (World Health Organisation [WHO], 2014), threatening delivery of effective healthcare and challenging basic procedures used in modern human and veterinary medicine (O’Neill, 2016).

In Europe, since 2006, non-therapeutic use of antimicrobials has been limited in livestock (Cogliani et al., 2011). However, concerns remain that antibiotic use in food animals for therapeutics increases the risk of selection and emergence of AMR bacteria; and animals are a reservoir (Argudin et al., 2017). Recent livestock studies in Great Britain (GB), suggest that *E. coli*, chosen as an indicator organism for monitoring AMR in commensal bacteria in the gut flora of livestock, may carry resistance to high priority critically important antibiotics (HP-CIAs) such as cefotaxime or colistin (Anjum et al., 2011; Randall et al., 2014; Card et al., 2016; Duggett et al., 2017; Kirchner et al., 2017), that may spread horizontally to pathogens. Harmonized monitoring of AMR across Europe has indicated resistance of *E. coli* to HP-CIAs varies from country to country in humans, animals, and food (European Food Safety Authority [EFSA], and European Centre for Disease Prevention and Control [ECDC], 2017); but typically only phenotypic analysis of AMR is performed on isolate(s) from a single representative animal on farm, recovered from selective and/or non-selective media. Recently, the fecal resistome of pigs and poultry from nine European countries was assessed using metagenomics, to define the on-farm AMR load (Munk et al., 2018). The study reported a higher AMR load in pigs than poultry, however, the organisms harboring the AMR genes was not defined, possibly due to limits of Illumina short read sequencing. Also, mobile genetic elements such as plasmids that play a key role in AMR dispersal could not be classified further.

The aim of this work was to characterize, using WGS, the AMR genes present in 492 *E. coli* from multiple pigs on farm, collected in a cross-sectional study, across GB. WGS also enabled phylogenetic diversity of the host *E. coli* to be explored, as well as putative AMR plasmids, which may play a key role in the transferring of AMR genes between *E. coli*. Presence of MDR isolates was analyzed with on-farm risk factors to identify features that affect their selection, as well as those that may help control their spread.

## Materials and Methods

### Farm Recruitment and Sampling

Fifty-six farms in GB were recruited, from two different production types (38 Farrow-to-Finish and 18 Finisher-only farms). Farms were geographically diverse, and loosely representative of the finisher-only pig industry in GB; no farms were selected from the North West, Wales or Scotland (Supplementary Table S1) (Pig Health and Welfare Council, 2017). The farms selected were not random but an opportunistic selection of 53 farms participating in a *Salmonella* study, and these included 19 farms with low prevalence of *Salmonella* (Martelli et al., 2017; Smith et al., 2017, 2018). Three additional farms were recruited solely for this study. All farmers provided written consent to allow their pigs to be sampled and their laboratory results used for research purposes. Sampling of farms took place at 12 different abattoirs, between March 2014 and October 2015. A comprehensive questionnaire, completed by farmers, collected information on farming practices that included hygiene and disinfection of houses, biosecurity, as well as antibiotic usage. Ethical approval was not sought as sampling from carcases is deemed outside of the Animal (Scientific Procedures) Act 1986.

### Sampling and Isolate Collection

At abattoirs, cecal content from 10 randomly selected healthy finishing pigs per herd/farm, were collected. For each herd, 0.5 g of pig cecal content from each pig was suspended into 22.5 ml of 0.1 M PBS (pH7.2) and the pooled pig cecal samples diluted up to 10^–5^ in PBS.

Aliquots of 100 μl were plated on to the following agar; Brilliance UTI agar (Oxoid, Basingstoke, United Kingdom) plates containing either 1 mg/L cefotaxime, 1 mg/L ciprofloxacin, and without antibiotics. Six morphologically distinct colonies were selected from each plate, however, if less than six colonies were isolated on either 1 mg/L cefotaxime or 1 mg/L ciprofloxacin plates, additional colonies from antibiotic-free plates were selected, to reach 18 isolates per farm. In addition, Brilliance carbapenem-resistant Enterobacteriaceae (CRE) Agar (Oxoid, Basingstoke, United Kingdom), was used. Purified colonies were subsequently stored at −80°C in MicroBank beads (Pro-Lab Diagnostics, Neston, Cheshire, United Kingdom). The presumptive purified *E. coli* were identified to species level using Matrix Assisted Laser Desorption Ionization-Time of Flight mass spectrometry (MALDI-ToF) (Bruker, Coventry, United Kingdom) or 16S rRNA sequencing (Edwards et al., 2012). Antimicrobial susceptibility testing was performed using the BSAC agar dilution method on the four *E. coli* isolated from CRE agar to determine susceptibility to three carbapenem antibiotics (Doripenem, Imipenem, and Meropenem), for all *E. coli* isolated from ciprofloxacin and cefotaxime antibiotic plates (Andrews, 2001). The results were interpreted using the European Committee on Antimicrobial Susceptibility Testing (EUCAST) epidemiological cut-off (ECOFF) and clinical breakpoint values.

### Whole Genome Sequence Analysis

DNA was extracted and Illumina HiSeq 4000 System used to perform WGS as described previously (Stoesser et al., 2013) on the 503 isolates identified as *E. coli* by MALDI-ToF. Kraken was used to confirm MALDI-ToF speciation results (Wood and Salzberg, 2014), which identified 11 isolates as *E. fergusonii*, a species that has been reported in livestock (Wragg et al., 2009), and excluded from further analysis. The presence of acquired AMR genes in the WGS of isolates was determined by mapping unassembled reads using APHA SeqFinder pipeline (Anjum et al., 2016). The criteria for determining gene presence using APHA SeqFinder pipeline was 100% gene mapping to the reference, and allowing between 1 and 10 non-synonymous SNP. The MIC results and the correlation between genotype and phenotype was >98% for *E. coli* isolates; these results have been presented elsewhere (Stubberfield et al., 2019), and due to the high correlation only the AMR genotype data is presented in this paper. Genotypic multidrug resistance was defined according to the European Food Safety Authority (EFSA) European surveillance reports (European Food Safety Authority [EFSA], and European Centre for Disease Prevention and Control [ECDC], 2017) as an isolate harboring genes from the following 3 or more resistance classes: Extended spectrum β-lactamase (ESBL), Ampicillin (AMP), Tetracycline (TET), Gentamicin - clinically relevant aminoglycoside (GEN), Azithromycin (AZM), Chloramphenicol (CHL), Trimethoprim (TMP), Sulphonamide (SUL), and Fluoroquinolones (FQN).

Sequenced genomes were assembled using SPAdes 3.7.0 11 (Bankevich et al., 2012) and annotated using PROKKA 1.11.12 (Seemann, 2014). The Abricate^1^ tool was used to determine which AMR and plasmid replicon genes were co-located on a single contig. BRIG was used to compare plasmids (Alikhan et al., 2011). The Multilocus Sequence Type (MLST) of *E. coli* isolates (Wirth et al., 2006) was determined using either SRTS2 (Inouye et al., 2014) or DTU pipeline^2^ (Larsen et al., 2012). SNIPPY^3^ was used to generate a whole genome single nucleotide polymorphisms (SNP) alignment produced from WGS data of 492 *E. coli* isolates using the default settings. A maximum-likelihood tree under the General Time Reversible model of nucleotide substitution with among-site rate heterogeneity model (GTR-G) and 100 bootstrap replicates was inferred from the whole genome of 272,385 SNP alignment using *E. coli* MG1655 (Accession number: U00096.2) as reference in RAxML-NG^4^. The tree was visualized and annotated in EvolView (He et al., 2016). The *E. coli* raw sequence data generated and analyzed in this work are available in the European Nucleotide Archive (ENA) under study accession number PRJEB26317.

### Statistical Analysis

The risk factor dataset contained 72 farm-level variables describing basic farm demographics, feeding and watering information including additive practices, and general farm management, which was provided to farmers for completion prior to cecal content sample collection and detection of the AMR gene content of isolates. Antimicrobial usage data were collected, however, due to difference in the detail of information provided by the farms, these responses were categorized subjectively into high, medium and low usage by the authors based upon answers to the total number of daily animal doses of antimicrobials given to pigs and the total cost of antimicrobials in a 12-month period. No standardized criteria existed to categorize the farms, but in general low usage farms were defined by an absence of prophylactic use of antibiotics and a rough cost of antibiotics under £0.5 per pig. Medium usage farms were defined by prophylactic use for short periods of time and a rough cost of antibiotics up to £2 per pig. High usage farms were generally either using in-feed or in-water treatment for multiple pig stages or using a combination of prophylactic/in-feed or in-water treatment and large quantities of injectables (e.g., routine injections to sows), with a rough cost of up to £3 per pig. The dataset also included a binary indicator of the MDR isolates gathered from WGS data analysis, for antimicrobials considered by EFSA (European Food Safety Authority [EFSA], and European Centre for Disease Prevention and Control [ECDC], 2017).

A simple descriptive assessment was completed of each variable, excluding variables with little or no data and creating new categorical variables, where appropriate. Following this, a univariable analysis utilizing a mixed-effects logistic regression model, accounting for the non-independence of multiple isolates per farm, was carried out to determine variables for inclusion (*p*-value < 0.25) in the multivariable model. Finally, to assess the effect of including variables at the multivariable level, an iterative, forward step-wise approach was adopted with variables that improved the model fit [assessed via *p*-value and Akaike Information Criterion (AIC) (Akaike, 1974)], were selected at each step of the model. The categorical variable, related to antimicrobial usage on each farm over a 12-month period, was classed as *a priori* and automatically retained in the model as was a variable accounting for the use of the four different agar plates used in the study. A *p*-value of less than 0.05 was considered to indicate a statistically significant difference. Potential confounding between variables were investigated by monitoring whether the addition of variables at each step inflated the Odds Ratio of the variables retained in the model. All statistical analyses were completed in Stata 12 (StataCorp, 2011).

## Results

### *E. coli* AMR Genes and MDR

**FIGURE 1:**
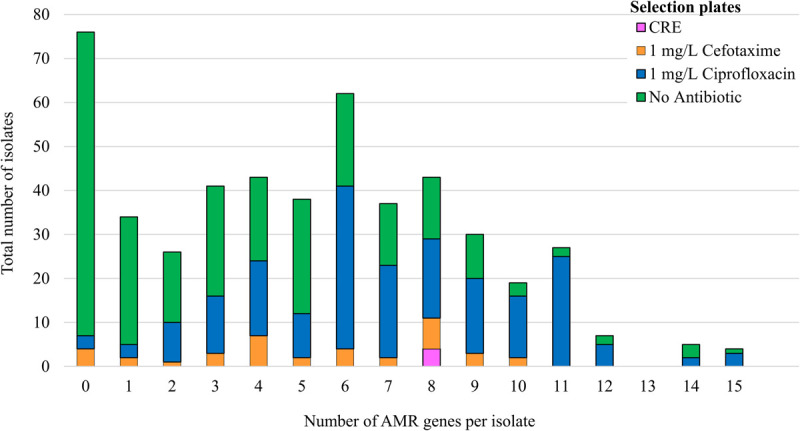
Correlation of AMR genes harbored by isolates with the selection plate. The number of AMR genes detected by WGS in 492 isolates from 56 pigs farms. The colored bars indicate the number of isolates selected on agar supplemented with: antibiotic-free plate (green); 1 mg/L ciprofloxacin (blue); 1 mg/L cefotaxime (orange); or from Brilliance carbapenem -resistant Enterobacteriaceae Agar plates (CRE; pink).

**FIGURE 2:**
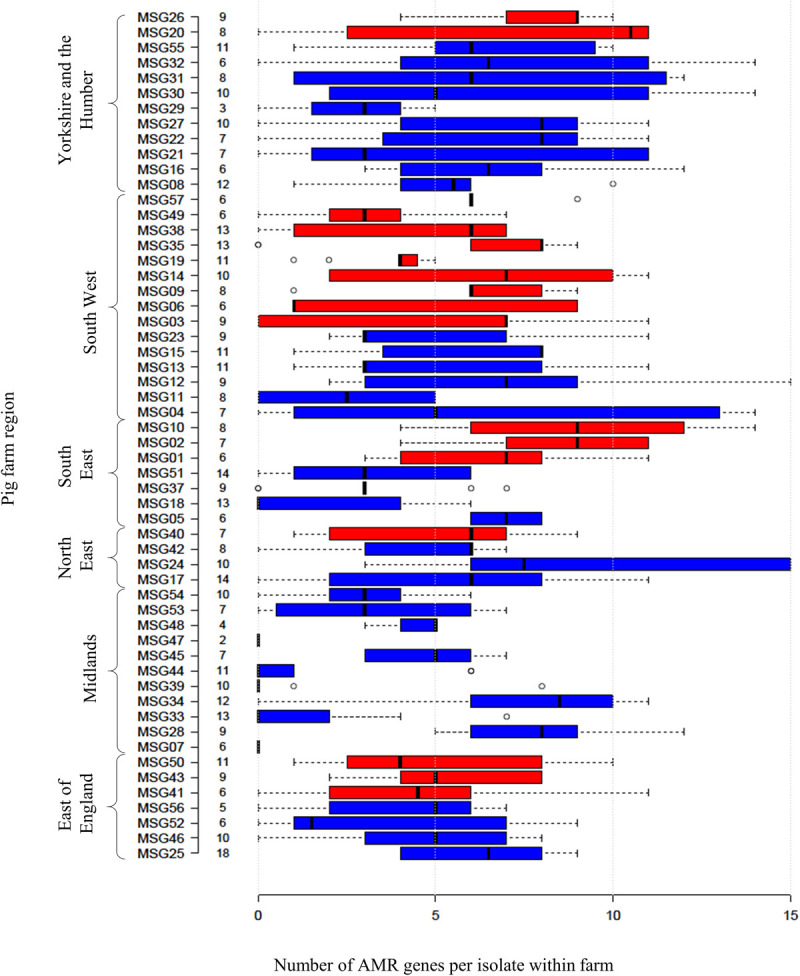
Relationship between numbers of AMR genes, farm region, and production type. Box plot representing the interquartile range or midspread of AMR genes found in isolates from a farm (numbered as MSG01–MSG35 and MSG37–MSG57). Farms were ordered according to their region and production type (farrow-to-finisher = blue and finisher-only = red). Number to left of bars indicate the number of isolates from farm. Center lines show the median; box limits indicate the 25th and 75th percentiles as determined by R software. Whiskers extend 1.5 times the interquartile range from the 25th and 75th percentiles, with outliers represented by dots.

Four hundred and ninety-two *E. coli* were recovered from 56 pig farms; 51.6% were recovered on non-selective plates and the remainder on antibiotic selective plates (Table 1). The APHA SeqFinder pipeline (Anjum et al., 2016) identified 62 different AMR gene variants in 84.6% (416 of 492) of the *E. coli* isolated which were from 54 farms (Table 1). For the remaining two farms, which were both farrow-to-finish farms, the *E. coli* did not harbor any AMR genes. For the remaining farms, *E. coli* harbored between 1 and 15 AMR genes (Figure 1), and as expected, isolates from the antibiotic-free plate generally had less AMR genes (average = 4.9) than those from antibiotic containing plates (average = 7), indicating co-selection of multiple resistances. The number of AMR genes present in *E. coli* from each farm was variable with the mean number of AMR genes per isolate per farm being 5.1 (Figure 2); there was no association between farms and the presence of specific AMR genes. The most common AMR genes were the tetracycline resistance genes [*tet(A)*], followed by β-lactamases (*bla*_TEM–1b_) and the streptomycin resistance genes (*strAB*) (Table 1). Only 9.3% of isolates harbored genes conferring resistance to clinically relevant aminoglycosides (gentamicin, amikacin, netilmicin, and tobramycin), and no 16S rRNA methyltransferase enzyme (16S RMTase) genes were identified (Magiorakos et al., 2012; Public Health England, 2017). Fourteen farms had *E. coli* isolates harboring two *bla*_TEM_ variants, with isolates from farm MSG54, harboring three different variants.

**TABLE 1:** Summary of antimicrobial resistance genes from different classes identified in *Escherichia coli* isolates.

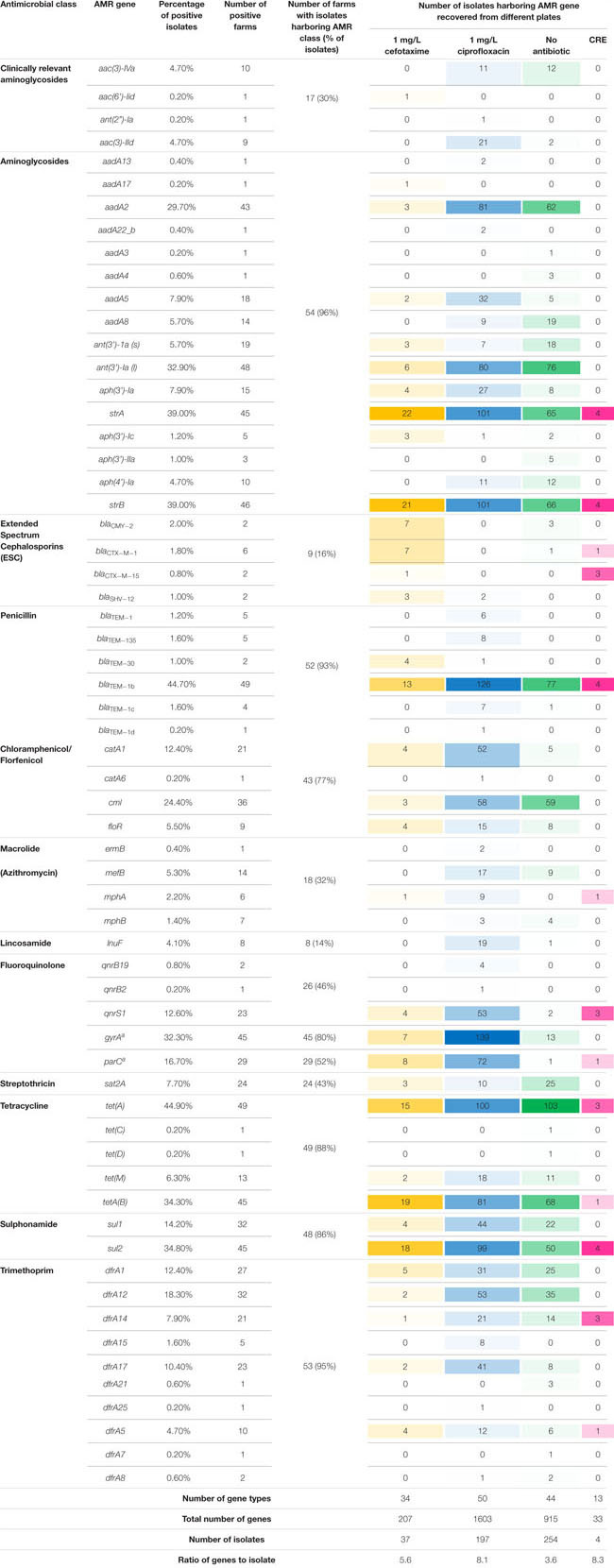

*^*a*^SNPs within QRDR in gyrA or parC resulting in non-synonymous amino acid mutations which result in antibiotic resistance.*

National (Public Health England, 2017) and European (European Food Safety Authority [EFSA], and European Centre for Disease Prevention and Control [ECDC], 2017) surveillance programs monitor AMR in commensal and pathogenic bacteria, mainly at the phenotypic level. To facilitate comparison between these datasets with our AMR genotypes, we queried genes present in the AMR classes included in these programs (Supplementary Table S2). Using this criterion, 35% of *E. coli* recovered on non-selective plates, from 40 farms, were genotypically MDR (Supplementary Figure S1). A higher proportion of isolates (87%) recovered on antibiotic selective plates, from 49 farms, were genotypically MDR. Sixty-four MDR genotypes were found; the most common genotype (ampicillin, tetracycline, sulphonamide, trimethoprim, and fluoroquinolone) was present in isolates from 12 farms in both production systems (Supplementary Table S3). Forty-eight MDR profiles (221 isolates) included resistance classes reported in *E. coli* from human blood infections such as ESBL, fluoroquinolone or clinically relevant aminoglycosides (Public Health England, 2017); however, no isolate harbored all 3 classes. Fifteen isolates harboring ESBL and fluoroquinolone resistance were detected on five farms.

We considered the linkage of AMR genes and the plasmid incompatibility types (Inc-types) by observing the co-location of these genes on the same contigs, using the *de novo* assembled genomes of isolates. Most isolates harbored between 1 and 7 (average 3) Inc-types, with 90 Inc-types and AMR gene combinations being present (Supplementary Table S4). The most common contigs harboring an AMR gene and plasmid Inc-types ranged in size from ∼4 to >100 Kb (Table 2); several of these contigs showed similarity to plasmids of diverse origins, mostly reported from *Salmonella* and *E. coli*. The number of farms and isolates in which these putative plasmid sequences were identified varied, with some (e.g., IncQ1, IncF, and IncI) present in isolates with different STs and farms. The putative IncQ1 plasmid harboring *strAB* and *sul2* was the most common and highly conserved, found in 48 isolates, but it only showed ∼50% identity to the closest references available in databases (Supplementary Figure S2).

**TABLE 2:** Plasmids involved in AMR gene dissemination.

**Plasmid Inc type**	**AMR genes associated with replicon type^a^**	**Other genes found in isolates^b^**	**Conitg size (Kb)^c^**	**Matches in NCBI database^d^ (accession number)**	**Bacterial species for matched plasmid^d^**	**Sample origin^e^**	**% alignment^f^**	**Number of Isolates**	**Number of farms**	**Number of MLST STs**
IncQ1	*strAB*, *sul2*	*repAC mobB*	4.4–4.7	pRSF1010 (NC001740)	*E. coli*	NK	53% (50–58%)	48	9	9
IncI1	*qnrS1*		18–106	pSTM2 (KF290378)	*Salmonella enterica* subsp. *enterica serovar Typhimurium*	Human	79% (75–82%)	19	6	7
IncFII	*strAB, bla*_TEM–1_, *sul2*, *tet(A)*, and *dfrA14*	*intI*, *tra* and *trb*	48	pM160133-p2 (CP022166)	*E. coli*	Human (urine)	95% (95–96%)	9	2	ST101
Unknown	*bla*_CTX–M–15_, *bla*_TEM–1_, *qnrS1*, *strAB*, and *sul2*		21–36	pAR0162 (CP021681) pEco-CTXM15 (MF510423)	*E. coli*	NK Human (bile)	80% (73–95%) 92%	3	2	2
IncI1	*bla*_CTX–M–1_, *sul2*	*tet(A)*	107	p369 (IncI) (KT779550)	*E. coli*	Chicken	99.7%	1	1 (MSG43)	ST3205
unknown	*bla*_CTX–M–1_, *sul3*, *dfrA1*	*intI*	85				91%	1	1 (MSG17)	ST101
IncA/C2	*bla*_CMY2_, (*sul2*)	*floR*, *tet(A)*, s*trAB*	127-128	pSN254b (KJ909290)	*Aeromonas salmonicida*	Fish farm	92%	2	1 (MSG25)	ST162
IncHI2A	*strAB*, *bla*_CMY2_	Tellurite	124	pYD786-1 (KU254578)	*E. coli*	Human (urine)	82% (81–84%)	4	1 (MSG25)	ST101
IncI1	*bla*_CMY2_		86	p85 (CP023362)	*E. coli*	Canine	99%	1	1 (MSG53)	ST156

*Summary of plasmids contigs present in isolates, as identified from WGS. ^*a*^AMR genes identified using APHA SeqFinder from WGS. Genes that are variably present are in parenthesis. ^*b*^Other genes found in isolate but not located on contigs harboring plasmid replicon. ^*c*^Size of resolved contigs harboring AMR gene and plasmid replicon type. ^*d*^Plasmid and bacteria with highest BlastN identity in NCBI database. ^*e*^Sample origin of bacterial species harboring matched plasmid. ^*f*^Percentage of aligned bases to reference calculated using Snippy.*

### Resistance to HP-CIAs in *E. coli*

Only 28 *E. coli* isolates (18 from cefotaxime plates) harbored genes, which mediate resistance to extended-spectrum cephalosporins (ESC) (Table 1); three farms had *E. coli* harboring two ESC resistance gene variants (*bla*_CTX–M–1_ and *bla*_CTX–M–15_, *bla*_CMY–2_ and *bla*_CTX–M–15_, or *bla*_CMY–2_ and *bla*_SHV–12_). ESC resistance genes were found co-located on contigs with plasmid replicons, with some showing high homology to published plasmids (Table 2). For example isolates from farm MSG17 and MSG43 harbored *bla*_CTX–M–1_ on a plasmid with high identity to the p369 IncI plasmid (Table 2 and Supplementary Figure S2). An additional 16 *E. coli*, isolated from cefotaxime plates, harbored mutations in the chromosomal AmpC promoter region associated with increased β-lactamase expression (Caroff et al., 2000).

Chromosomal mutations in quinolone resistance determining regions (QRDR) of *gyrA* and/or *parC* (Yoshida et al., 1991) were identified in 72.6% of *E. coli* (from 44 farms) recovered from ciprofloxacin plates (Table 1), with MICs ranging from 2 to 64 mg/L; 53.1% of these isolates were clinically resistant to ciprofloxacin (>4 mg/L). Only 8.5% of *E. coli* (from 18 farms) recovered from the other agar plates harbored mutations in QRDR, with MICs ranging between 2 and 128 mg/L; 48% of these were clinically resistant to ciprofloxacin (Table 1). Of the QRDR mutations identified, S83L/D87N double substitution (*n* = 80) was the most common for GyrA, and S80I substitution (*n* = 80) most common for ParC; 73 of these isolates harbored double-serine mutation (Fuzi et al., 2017), and the majority were recovered on ciprofloxacin plates (Supplementary Table S5). In addition, three plasmid mediated quinolone resistant (PMQR) variants (Redgrave et al., 2014) were detected in 67 *E. coli* (13.6%) with the most common gene being *qnrS1* (Table 1). Majority of *E. coli* (*n* = 56) harboring PMQR genes did not harbor QRDR mutations; the MICs levels ranged between 0.06 and 8 mg/L, with only four isolate showing clinical resistance (>1 mg/L). Nineteen isolates had a contig encoding both *qnrS1* and IncI (Table 2), which showed ∼79% sequence identity to the pSTM2 plasmid found in *Salmonella* Typhimurium (Supplementary Figure S2), which may be present in these isolates.

Four *E. coli* from two farms, recovered on carbapenem selection media, did not harbor transmissible carbapenem resistance genes and were phenotypically susceptible to carbapenems (Supplementary Table S6); these isolates were highly resistant to cefotaxime which may have contributed to the growth on CRE plates (MIC > 32 mg/L). Plasmid mediated colistin resistance genes were not detected.

### Phylogenetic Diversity

**FIGURE 3:**
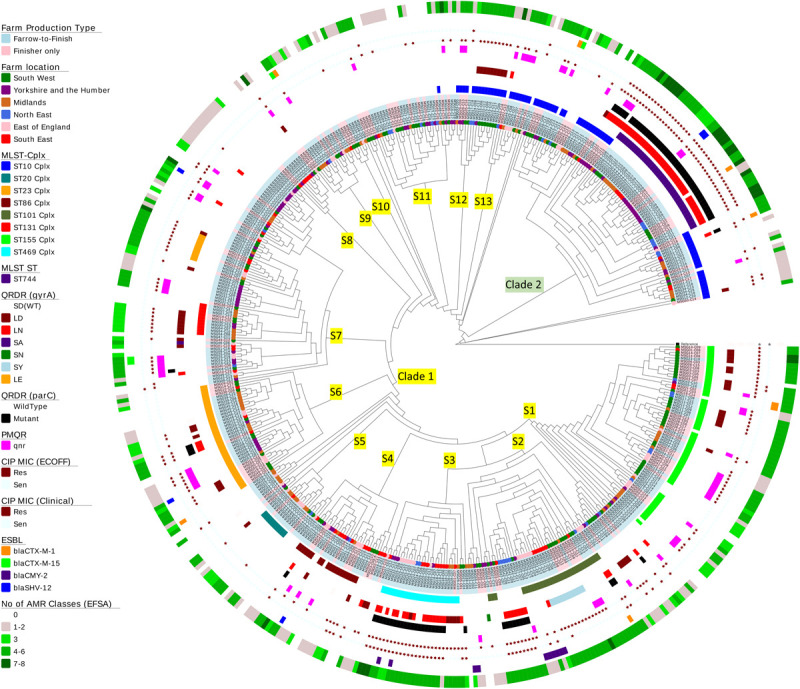
Phylogenetic tree of *Escherichia coli* isolates. A SNP-based maximum-likelihood phylogenetic tree was constructed in RaxML-NG using 272,385 single nucleotide polymorphisms (SNP) present in the core genome. Clades and subclades are labeled on the relevant branches. Isolate names are colored according to farm production type (blue, farrow-to-finish; pink, finisher-only) and nodes at the end of the leaves are colored according to the six regions in England from where the *E. coli* isolates originate. The circles at the edge indicate the following information (working from inner circle to outer circle): (1) the top 8 MLST clonal complexes identified; (2) MLST ST744; (3) QRDR mutations in *gyrA*; (4) QRDR mutations in *parC*; (5) presence of plasmid quinolone resistance (*qnr* genes); (6) ciprofloxacin susceptibility using EUCAST ECOFF; (7) ciprofloxacin susceptibility using EUCAST clinical breakpoints; (8) presence of ESBL gene variants; and (9) the numbers EFSA AMR classes harboring different resistance genes in each isolate (genes conferring resistant to the same antimicrobial were grouped within the same class).

A whole genome SNP-based maximum-likelihood phylogenetic tree was constructed and separated isolates into two distinct clades (1 and 2) with the majority of isolates (*n* = 394) clustering within clade 1 (Figure 3). There was little evidence of clustering by phylogeography, production type or farm, with the only exception being farm MSG31, where all isolates clustered within S7.

*Escherichia coli* isolates belonged to 117 multilocus sequence types (MLST) which included 19 clonal complexes and 17 new ST variants. Nearly half the isolates belonged to seven STs, but there were no apparent association between AMR profiles and ST (Figure 3 and Supplementary Table S7). On average, farms had *E. coli* from five STs, with the exception of farm MSG17, which harbored nine STs. Fifty-eight percent of isolates, from different geographic regions, were located in clade 2; the majority were ST744, which has one SNP variation from ST10. The remainder clustered in three sub-clades within clade 1 and majority of isolates (*n* = 15) belonged to ST162 (ST469 complex). Sixty-five percent of the STs (*n* = 76) identified have previously been observed in human *E. coli* (Supplementary Table S7), however, due to lack of information in the MLST database it is not clear whether these are of clinical origin. Nevertheless, 89 isolates belonged to seven STs commonly associated with extra-intestinal pathogenic *E. coli* (ExPEC) from United Kingdom bacteraemia and UTI in man (Gibreel et al., 2012; Manges and Johnson, 2012; Horner et al., 2014; Kallonen et al., 2017; Day et al., 2019), which included ST10, ST117, and ST131 (Supplementary Table S7). Ten ST131 *E. coli*, from four farms, clustered within clade S7. However, unlike MDR human ST131 isolates (Nicolas-Chanoine et al., 2014) they were phenotypically susceptible at clinical breakpoints to ciprofloxacin (Figure 3); although seven ST131-*fimH22* isolates from 3 farms harbored a single GyrA SNP (S83L), which led to resistances above the ECOFF and these isolates were MDR but not to any other HP-CIA; three ST131-*fimH298* isolates, all from the same farm, had no detectable AMR genes. Forty-seven of the 79 isolates (60%) from the remaining six STs showed a MDR genotype which included resistance to HP-CIAs (15 harbored resistance to gentamicin, 5 to ESBLs and 31 to fluoroquinolone, although none harbored resistance to all three classes to HP-CIAs).

Clustering was noted based on mutations in *gyrA* and *parC*. Half the isolates within clade 2 (*n* = 42) harbored double *gyrA* (S83L/D87N or LN) and a *parC* QRDR mutations, as did a number of isolates primarily from sub-clades 2 and 3 within clade 1 (7.6%, *n* = 30). Seventy-one isolates harboring both LN and *parC* mutations were clinically ciprofloxacin resistant; 94% (*n* = 67) of these were genotypically MDR (Figure 3).

### On-Farm Risk Factors

Multivariable risk factor analysis was performed at sample level rather than farm level using information gathered in a detailed questionnaire on farming practices, including hygiene and disinfection of houses, biosecurity, as well as antibiotic usage; the type of agar MDR isolates were selected on was also included. There were 29 potential risk factors (*p* < 0.25) identified at univariable screening but the final multivariable logistic regression model retained only six risk factors associated with farms harboring genotypically MDR *E. coli* (Table 3). The retained variables included those identified as *a priori* (antimicrobial usage and ciprofloxacin enrichment plate) and four additional variables selected by forward stepwise selection that were significantly (*p* < 0.05) associated with the outcome and provided the best model fit (indicated by a lower AIC). Not surprisingly, the greater the antibiotic usage on farms, the greater the risk of MDR *E. coli*. Farms with a medium usage of antimicrobial products had almost three times the odds of MDR than farms with low usage (*p* = 0.050), whereas farms with high usage of antimicrobial products had over five times the odds of MDR (*p* < 0.001). Isolates recovered from ciprofloxacin plates had a higher probability of being MDR compared to isolates from the cefotaxime and non-selective plates (both *p* < 0.001); the carbapenem-resistant Enterobacteriaceae plate had too few records to produce a credible result.

**TABLE 3:** Multivariable risk factor analysis, assessing for the association between genotypic multi-drug resistance and variables of interest (*n* = 492, of which 296 were multi-drug resistant).

**Variable**	**Level**	**No. isolates**	**Odds ratio**	***P*-value**	**95% confidence intervals**	
Antimicrobial usage	Low	72	*Baseline*			
	Medium	116	2.84	0.050	1.00	8.08
	High	151	7.64	<0.001	2.73	21.35

Antibiotic selective or non-selective plate^#^	1 mg/L CIP	197	*Baseline*			
	1 mg/L CTX	37	0.15	<0.001	0.06	0.40
	CRE*	4	Undefined	0.985	–	–
	No antibiotic	254	0.03	<0.001	0.02	0.07

Production type	Breeder-finisher	339	*Baseline*			
	Finisher only	153	3.69	<0.001	1.79	7.58

How often is manure pit emptied	Weekly	65	*Baseline*			
	Monthly	108	2.09	0.114	0.84	5.20
	Per batch	123	3.57	0.023	1.20	10.65
	Half yearly	8	0.46	0.513	0.05	4.67

Region of farm	South West	137	*Baseline*			
	South East	63	3.65	0.012	1.32	10.07
	East of England	65	0.60	0.251	0.25	1.44
	Midlands	91	1.11	0.821	0.44	2.85
	Yorkshire and Humber	97	0.42	0.038	0.19	0.95
	North East	39	1.66	0.381	0.53	5.15

On average, how often are pens cleaned and disinfected	Always, sometimes or between batches	413	*Baseline*			
	Never	43	0.27	0.012	0.10	0.75
	Missing	36	1.24	0.708	0.40	3.80

Variable levels for ‘not known’ or missing answers have been omitted from the table. *Small study population led to large uncertainty for odds ratio. ^#^CRE, carbapenem-resistant Enterobacteriaceae; CIP, ciprofloxacin; CTX, cefotaxime.

Specialist finisher farms were at a higher risk of MDR than breeder-finisher farms (*p* < 0.001), while those farms that emptied their manure pit after every batch were at greater risk than those that emptied it weekly (*p* = 0.023). Isolates from farms that on average, never cleaned and disinfected finisher pens between batches of pigs, were less likely to have MDR detected (*p* = 0.012). This answer was only provided by breeder-finisher farms and might have been due to continuous production; variables related to this factor did not appear to effect the selection of this factor, indicating that they were unlikely to fully explain this outcome. The geographical region of the farm was also selected as a significant factor, with farms in the South East being at lower risk of MDR (*p* = 0.012) and farms in Yorkshire and the Humber region at higher risk (*p* = 0.038) than those in the South West.

## Discussion

This study aimed to characterize the AMR genes, using WGS, present in 492 *E. coli* from pig farms in GB. The most common AMR genes harbored by these phylogenetically diverse *E. coli* of different STs were to “old” antimicrobials, including aminoglycosides (which were not within the clinically important group), tetracyclines, ampicillin, sulphonamides, and trimethoprim (Hopkins et al., 2007; Card et al., 2014, 2015; Kirchner et al., 2014; Ulstad et al., 2016). None of the porcine *E. coli* concurrently harbored resistance genes to ESBL, fluoroquinolone, and clinically relevant aminoglycosides, but 15 isolates from five farms carried AMR genes to the former two antimicrobials. This is in contrast to the AST data reported by Public Health England (PHE) for humans between 2012 and 2016 where ∼5% of *E. coli* isolated from blood and cerebrospinal fluid showed resistance to third-generation cephalosporin, fluoroquinolones, and aminoglycosides, specifically gentamicin and/or tobramycin, which nevertheless have previously been reported from livestock (Szmolka et al., 2012). Also, the 16S RMTase genes reported from human clinical isolates (Public Health England, 2017), were absent. Importantly, several *E. coli* STs that have been isolated from humans were present, including seven collected from bacteraemia or feces of people admitted to UK hospitals, indicating some overlap between these compartments; many of these isolates were genotypically MDR and included resistance to HP-CIAs. Of the 10 ST131 isolates, 7 harbored the *fimH22* fimbriae, progenitor of *fimH30* associated with ST131 human pathogens (Ben Zakour et al., 2016). A previous study in retail meat also found that H22 strains were common and suggested the potential importance of *E. coli* ST131-*fimH22* as a foodborne pathogen (Liu et al., 2018). While this study only examined *E. coli*, the repertoire of AMR genes is likely to be greater due to other bacteria present in the pig intestine; for example *Moraxella* species harboring *mcr* (AbuOun et al., 2017) and three methicillin resistant *Staphylococcus aureus* (Sharma et al., 2016) has been reported from pig samples collected in this study. However, a thorough survey of the diverse bacterial population within the pig intestine was beyond the scope of this work.

In this study, 35% of *E. coli* isolates recovered from non-selective plates were genotypically MDR. This is lower than the average reported across Europe (38.1%) and United Kingdom (51%) from non-selective media from pigs during 2015 (European Food Safety Authority [EFSA], and European Centre for Disease Prevention and Control [ECDC], 2017). Differences in the number of farms sampled, methods of collection and isolation, and numbers of isolates tested could account for the variations; this study included 18 isolates/farm compared to only one isolate/farm included in EU surveillance. In addition, we used WGS to determine presence of AMR genes, which is highly predictive of AMR presence and could be more sensitive than phenotyping (Ellington et al., 2017; Stubberfield et al., 2019).

Carbapenem usage is not permitted in food-producing animals in the United Kingdom (Grace et al., 2015; Broadfoot et al., 2016), therefore absence of plasmid mediated carbapenem resistance genes, a serious public health threat was reassuring (Logan and Weinstein, 2017). The sales/usage in animals of 3rd generation cephalosporins is low (Broadfoot et al., 2016), so the low percentage of farms (16%) positive for ESBL-harboring *E. coli* was expected; although it was lower than the figures reported from EU ESBL *E. coli* monitoring (EU prevalence = 31.9%, UK prevalence = 21.7%) (Randall et al., 2014; European Food Safety Authority [EFSA], and European Centre for Disease Prevention and Control [ECDC], 2017). No isolates harboring a *bla*_CTX–M_ group 1 enzyme belonged to ST73, ST131, or ST95, STs associated with human infections (Gibreel et al., 2012; Horner et al., 2014; Kallonen et al., 2017).

The highest level of resistance to HP-CIAs was seen in fluoroquinolones and multivariable analysis indicated significantly higher likelihood of *E. coli* harboring MDR being isolated from ciprofloxacin compared to cefotaxime or non-selective plates. Thus, ciprofloxacin plates enabled detection not only of ciprofloxacin resistant *E. coli*, most often with QRDR mutations, but additional AMR genes. Furthermore, these MDR *E. coli* were less likely to be detected on other plates because they probably represented a small percentage of the total *E. coli*, as previously shown for *mcr-*harboring *E. coli* isolated from pig farms (Randall et al., 2018). QRDR mutations in *Salmonella* can confer protection against other antimicrobials (Webber et al., 2013) and the presence of double-serine QRDR mutations (GyrA83Ser and ParC80Ser) confers a selective advantage in lineages of several bacterial pathogens (Fuzi et al., 2017). We speculate these mutations may also aid on-farm persistence of QRDR *E. coli* enabling them to gain MDR status by acquiring more AMR plasmids. It could then lead to increased antibiotic use due to possible treatment failures, as supported by multivariable analysis, which showed increased risk of MDR *E. coli* being present on farms with high antibiotic usage. Farmers should limit or cease in-feed or in-water antibiotic treatment of pigs at herd level in order to reduce the risk of MDR. More targeted treatment of individual pigs would be preferable, although this would only be suitable where any reduction in use did not impact upon the welfare of the pigs.

Additionally, improvements to biosecurity or more frequent emptying of the manure pit on farm may lead to reduced risk of MDR *E. coli* being present and hence less need for treatment. The frequent emptying of the pit below the flooring in pig housing has been recommended previously as a factor associated with reducing the prevalence of *Salmonella* on pig farms (Beloeil et al., 2004). Never using disinfectants on average when cleaning a pen was found to be protective, which may infer disinfectants are being diluted inappropriately. It is known that bacteria can become resistant to disinfection when exposed to sublethal concentrations (Zou et al., 2014). Finisher farms were shown to be at higher risk of MDR, which may reflect the differences in management (such as the number of incoming movements and sources used) and pig types present, when compared to breeder-finisher farms. The region of the farm was also shown to be associated with MDR, which may reflect the density of pig farm production in those significant areas, as Yorkshire and Humber is a known high farm-density area, whereas the South East is sparsely populated. Although in this study each farm was sampled in more depth than many other cross-sectional national study, including for the EFSA harmonized monitoring of AMR. These results should nevertheless be treated with some caution as only 10 pigs were sampled per farm, and only from healthy slaughtered pigs, so it may not have been fully representative of these farms and appropriate for all farm types, such as those with only breeding pig stock.

As plasmids play a role in the mobilization of AMR genes between bacteria (Frost et al., 2005), putative plasmid contigs were used as surrogates for detecting circulating AMR plasmids. Isolates harbored between one and seven Inc-types and the most commonly circulating AMR plasmids belonged to the broad host range IncQ family (Loftie-Eaton and Rawlings, 2012), followed by IncI plasmids, which is frequently associated with AMR dissemination (Rozwandowicz et al., 2018). Future studies performed with hybrid short and long-read sequencing of isolates will enable a more complete resolution of plasmid genomes and other mobile genetic elements (MGEs) to accurately define the regions flanking AMR genes, and their transmission. However, high homology of plasmid genomes from this study with others present in the databases illustrate plasmids are part of a global network, cycling and disseminating AMR genes, as already made apparent by the *mcr-*plasmid phenomenon (Wyrsch et al., 2016; Duggett et al., 2017, 2018; Figueiredo et al., 2019).

## Conclusion

We used genomics to detect MDR *E. coli* present on pig farms and combined it with multivariable analysis to identify factors affecting their selection, and possible control measures to help mitigate their transmission. Future studies can define common circulating plasmid genomes and other MGEs harboring AMR genes more fully and verify whether measures recommended to control their dissemination were successful.

### Limitations

The farmer questionnaires were not completed fully by all farmers. Many of the questions were in a free format answer, which made it difficult to standardize and compare the results between farms, for example, it was difficult to gage levels of antibiotics used by farmers. Classification of antimicrobial usage was subjective and may have led to some misclassification bias. For the risk factor analysis, the sample size was large enough to detect large associations (Odds Ratio >6.5) with 95% confidence and 80% power. However, as the number of pigs sampled on each farm was relatively low, and the number of *E. coli* isolates per farm in the analysis differed, the results may not be fully representative of the population on that farm. A random effect was utilized in the risk factor model to account for the differing levels of clustering of isolates from farms and their non-independence.

## Data Availability Statement

The datasets generated for this study can be found in the SRA PRJEB26317.

## Ethics Statement

APHA undertakes research using animals under the Animal (Scientific Procedures) Act 1986 (ASPA) which includes having it’s own Animal Welfare and Ethics Board (AWERB). The AWERB looks at all aspects of the Science Division’s use of animals. As we had approval from the owners of the animals and the animals sampled were dead, this was outside ASPA (which only concerns live animals) and so it was considered that ethical approval from the committee was not required. Written informed consent was received from all participating farmers to allow their pigs to be sampled and their laboratory results and questionnaire data to be used for research purposes.

## Author Contributions

MFA and RS conceived and directed the project and designed the work. MFA and DC funding acquisition. MA, ES, and EJS performed bacteriology laboratory work. MA, ES, EJS, and JN-G performed the gene and WGS analysis. MA, HO’C, EJS, and MFA interpreted the results. MA, RS, and MFA wrote the manuscript.

## Conflict of Interest

The authors declare that the research was conducted in the absence of any commercial or financial relationships that could be construed as a potential conflict of interest.

## Footnotes

1 https://github.com/tseemann/abricate

2 https://cge.cbs.dtu.dk/services/MLST/

3 https://github.com/tseemann/snippy

4 https://github.com/amkozlov/raxml-ng

## Abbreviations

AMR: antimicrobial resistance
CRE: Brilliance carbapenem-resistant Enterobacteriaceae agar
HP-CIAs: highest priority critically important antibiotics
MDR: multidrug resistance (resistance to 3 or more antimicrobial classes)
WGS: whole genome sequencing.
