# Corrigendum: Characterizing Antimicrobial Resistant *Escherichia coli* and Associated Risk Factors in a Cross-Sectional Study of Pig Farms in Great Britain

**DOI:** 10.3389/fmicb.2021.693940

**Published:** 2021-05-28

**Authors:** Manal AbuOun, Heather M. O'Connor, Emma J. Stubberfield, Javier Nunez-Garcia, Ellie Sayers, Derick W. Crook, Richard P. Smith, Muna F. Anjum

**Affiliations:** ^1^Department of Bacteriology, Animal and Plant Health Agency, Weybridge, United Kingdom; ^2^National Institute for Health Research, Health Protection Research Unit, University of Oxford in Partnership With Public Health England (PHE), Oxford, United Kingdom; ^3^Department of Epidemiological Sciences, Animal and Plant Health Agency, Weybridge, United Kingdom; ^4^Modernising Medical Microbiology Consortium, Nuffield Department of Medicine, John Radcliffe Hospital, University of Oxford, Oxford, United Kingdom

**Keywords:** antimicrobial resistance, multidrug resistance, plasmids, epidemiology, risk factor analysis, pig farms, *Escherichia coli*, Great Britain

In the original article, there was an error in the figure quoted in the abstract for the percentage of genotypically MDR *E.coli* (46.9% should be 35%) and percentage of ESBL *E. coli* (~4% should be ~2%) isolated from non-selective media.

A correction has been made to the abstract, sixth sentence. The corrected abstract is below:

Combatting antimicrobial resistant (AMR) using a One-Health approach is essential as various bacteria, including *Escherichia coli*, a common bacteria, are becoming increasingly resistant and livestock may be a reservoir. The AMR gene content of 492 *E. coli*, isolated from 56 pig farms across Great Britain in 2014–2015, and purified on antibiotic selective and non-selective plates, was determined using whole genome sequencing (WGS). The *E. coli* were phylogenetically diverse harboring a variety of AMR profiles with widespread resistance to “old” antibiotics; isolates harbored up to seven plasmid Inc-types. None showed concurrent resistance to third-generation cephalosporins, fluoroquinolones and clinically relevant aminoglycosides, although ~3% harbored AMR genes to both the former two. Transferable resistance to carbapenem and colistin were absent, and six of 117 *E. coli* STs belonged to major types associated with human disease. Prevalence of genotypically MDR *E. coli*, gathered from non-selective media was 35% and that of extended-spectrum-beta-lactamase *E. coli* was low (~2% from non-selective). Approximately 72.6% of *E. coli* from ciprofloxacin plates and only 8.5% from the other plates harbored fluoroquinolone resistance due to topoisomerase mutations; the majority were MDR. In fact, multivariable analysis confirmed *E. coli* purified from CIP enrichment plates were more likely to be MDR, and suggested MDR isolates were also more probable from farms with high antibiotic usage, specialist finisher farms, and farms emptying their manure pits only after each batch. Additionally, farms from the South East were more likely to have MDR *E. coli*, whereas farms in Yorkshire and the Humber were less likely. Future investigations will determine whether suggested improvements such as better biosecurity or lower antimicrobial use decreases MDR *E. coli* on pig farms. Although this study focuses on pig farms, we believe the methodology and findings can be applied more widely to help livestock farmers in the United Kingdom and elsewhere to tackle AMR.

The authors apologize for this error and state that this does not change the scientific conclusions of the article in any way. The original article has been updated.

