# Supplementary material for: Characterizing Antimicrobial Resistant Escherichia coli and Associated Risk Factors in a Cross-Sectional Study of Pig Farms in Great Britain
Source: Front Microbiol. 2020 May 25;11:861. doi: 10.3389/fmicb.2020.00861 (PMC7261845; doi:10.3389/fmicb.2020.00861)

## Slide 1
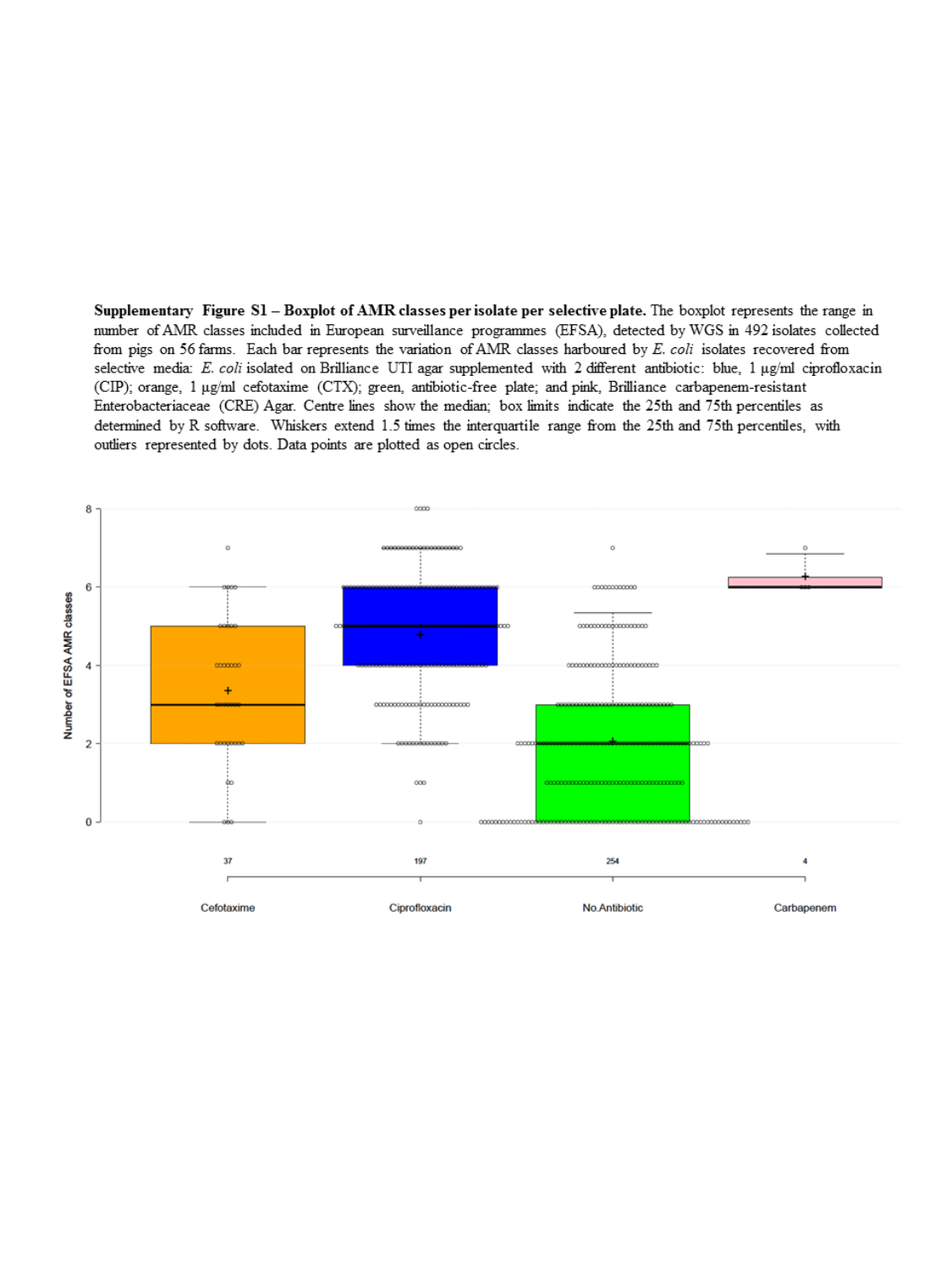

## Slide 2
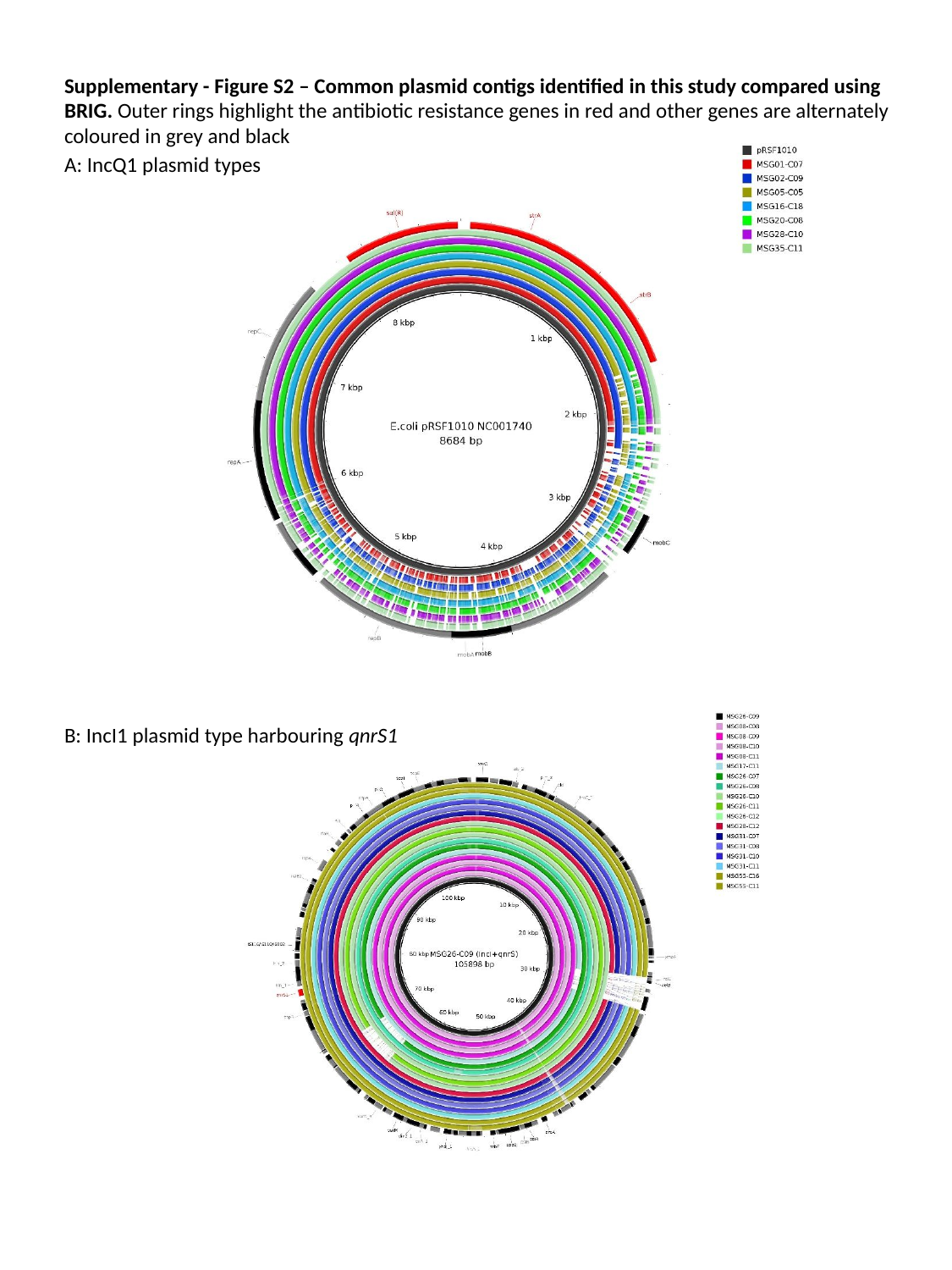

Supplementary - Figure S2 – Common plasmid contigs identified in this study compared using BRIG. Outer rings highlight the antibiotic resistance genes in red and other genes are alternately coloured in grey and black
A: IncQ1 plasmid types
B: IncI1 plasmid type harbouring qnrS1

## Slide 3
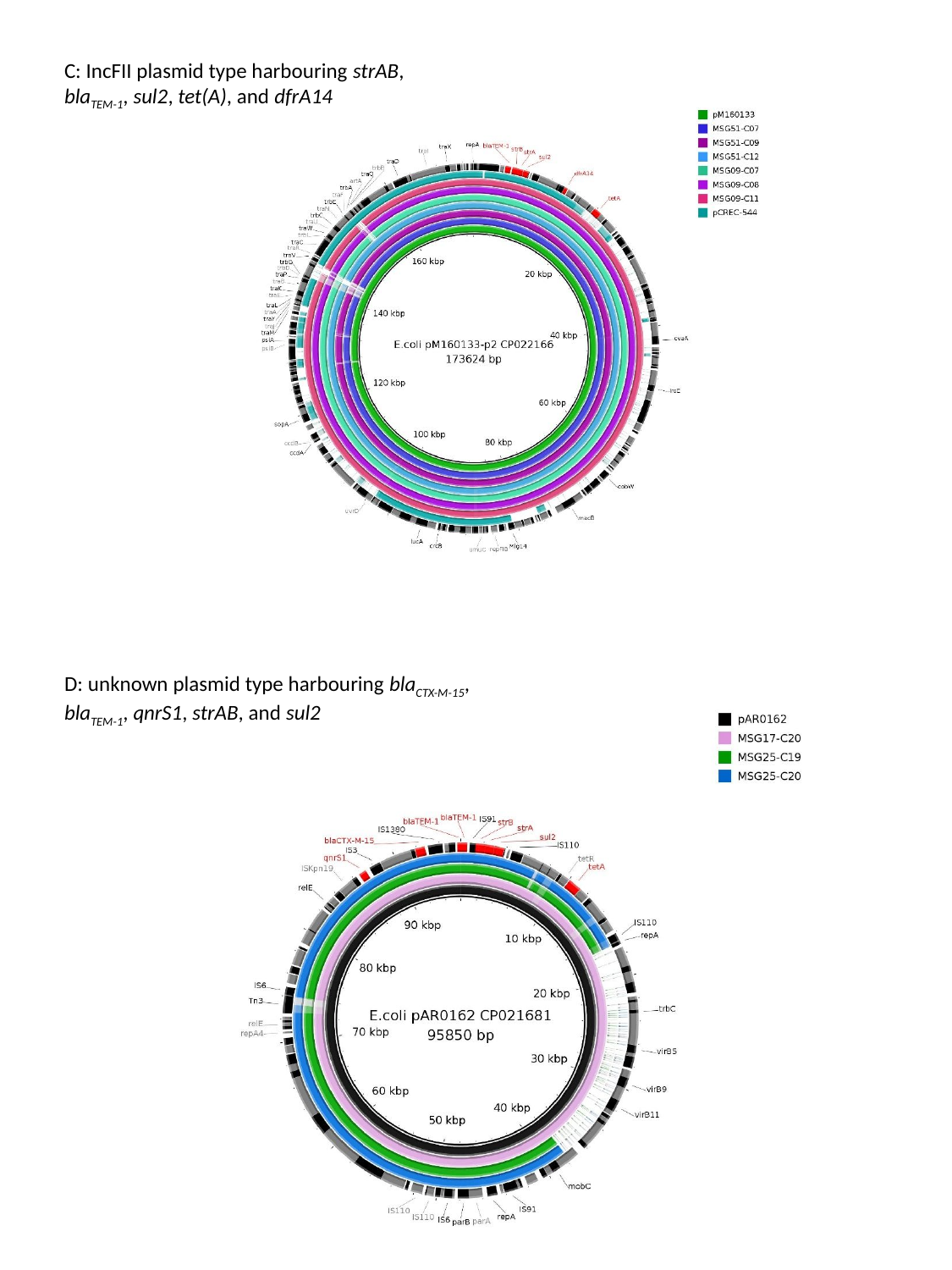

C: IncFII plasmid type harbouring strAB, blaTEM-1, sul2, tet(A), and dfrA14
D: unknown plasmid type harbouring blaCTX-M-15, blaTEM-1, qnrS1, strAB, and sul2

## Slide 4
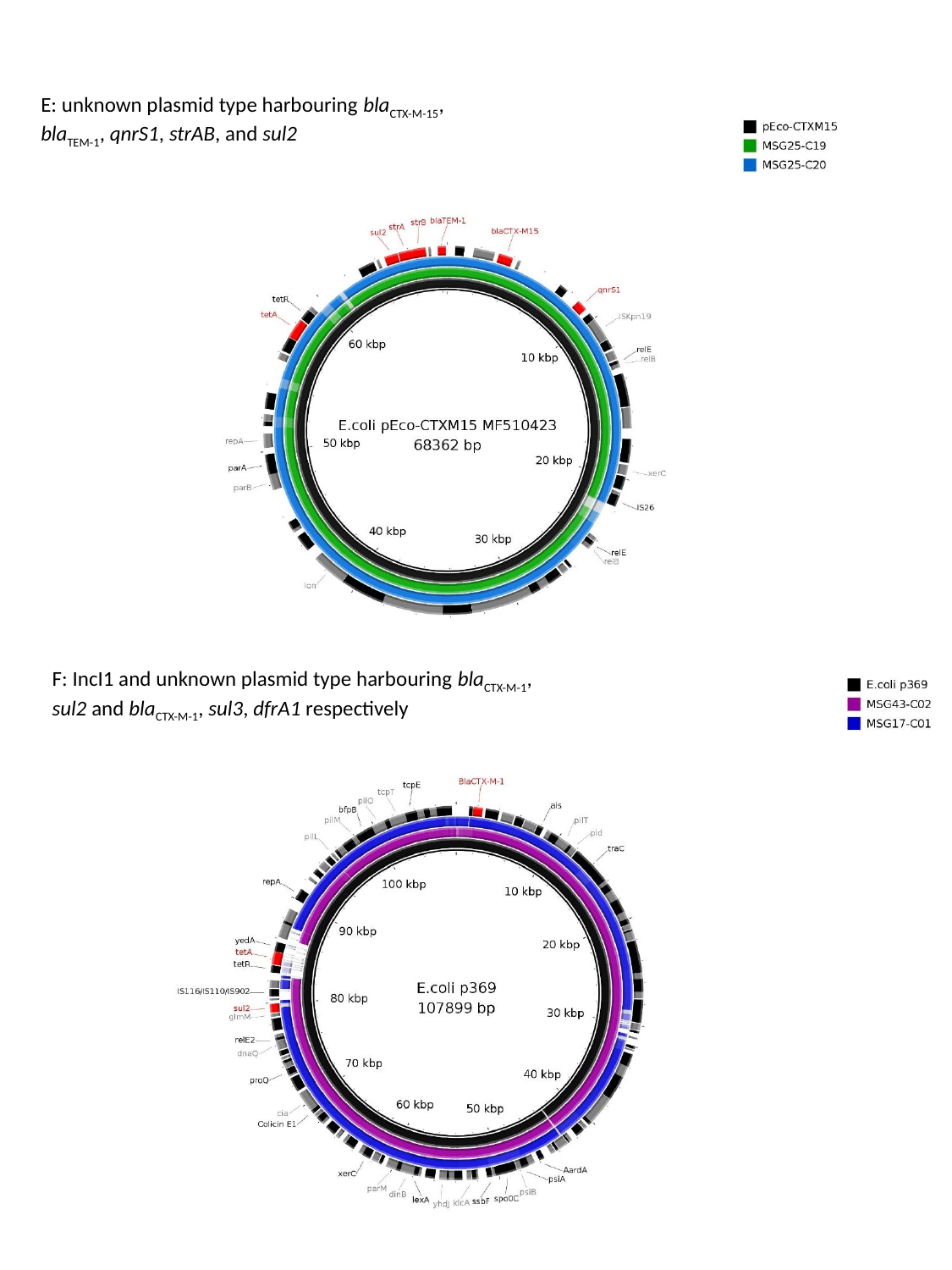

E: unknown plasmid type harbouring blaCTX-M-15, blaTEM-1, qnrS1, strAB, and sul2
F: IncI1 and unknown plasmid type harbouring blaCTX-M-1, sul2 and blaCTX-M-1, sul3, dfrA1 respectively

## Slide 5
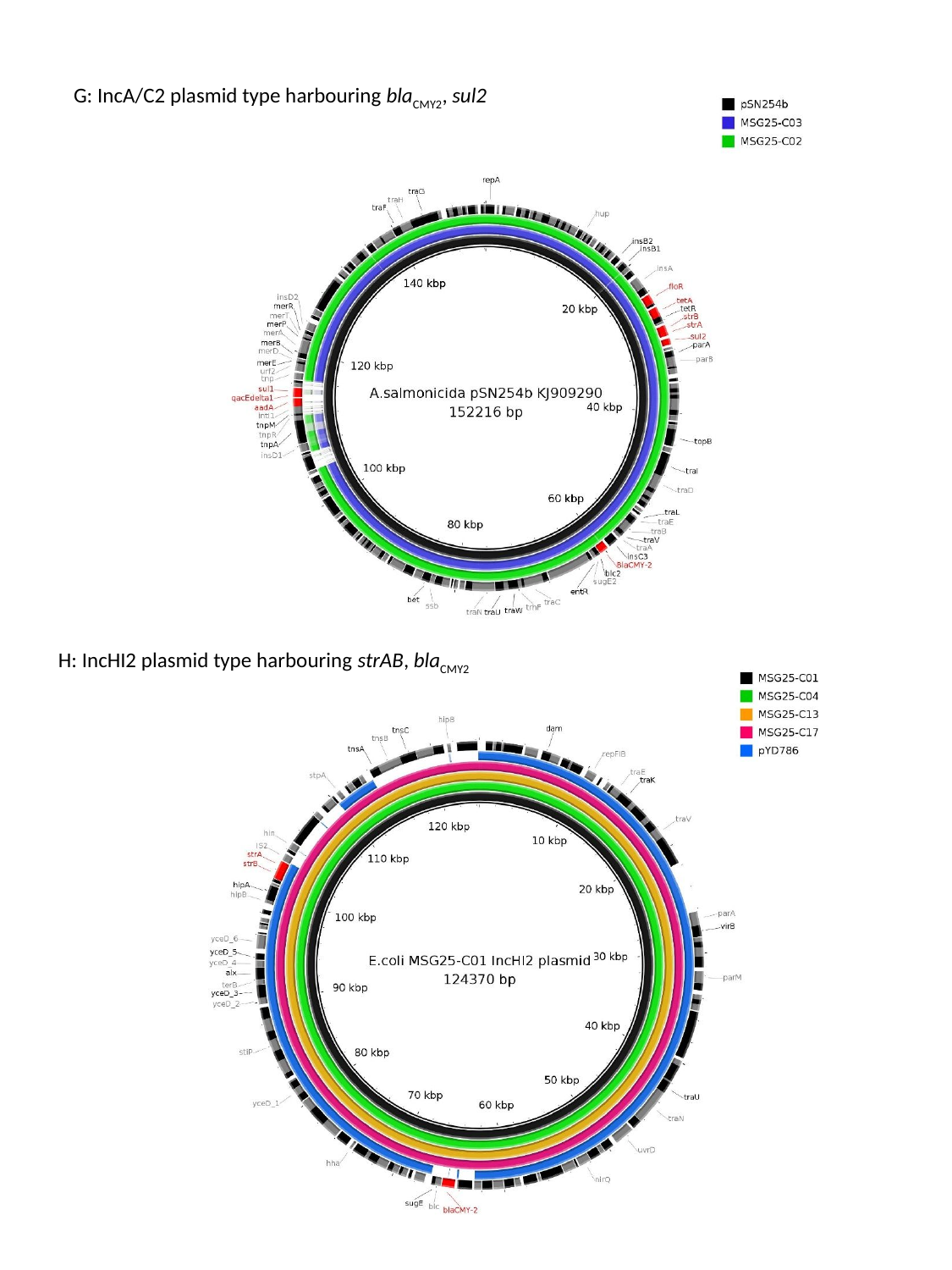

G: IncA/C2 plasmid type harbouring blaCMY2, sul2
H: IncHI2 plasmid type harbouring strAB, blaCMY2

## Slide 6
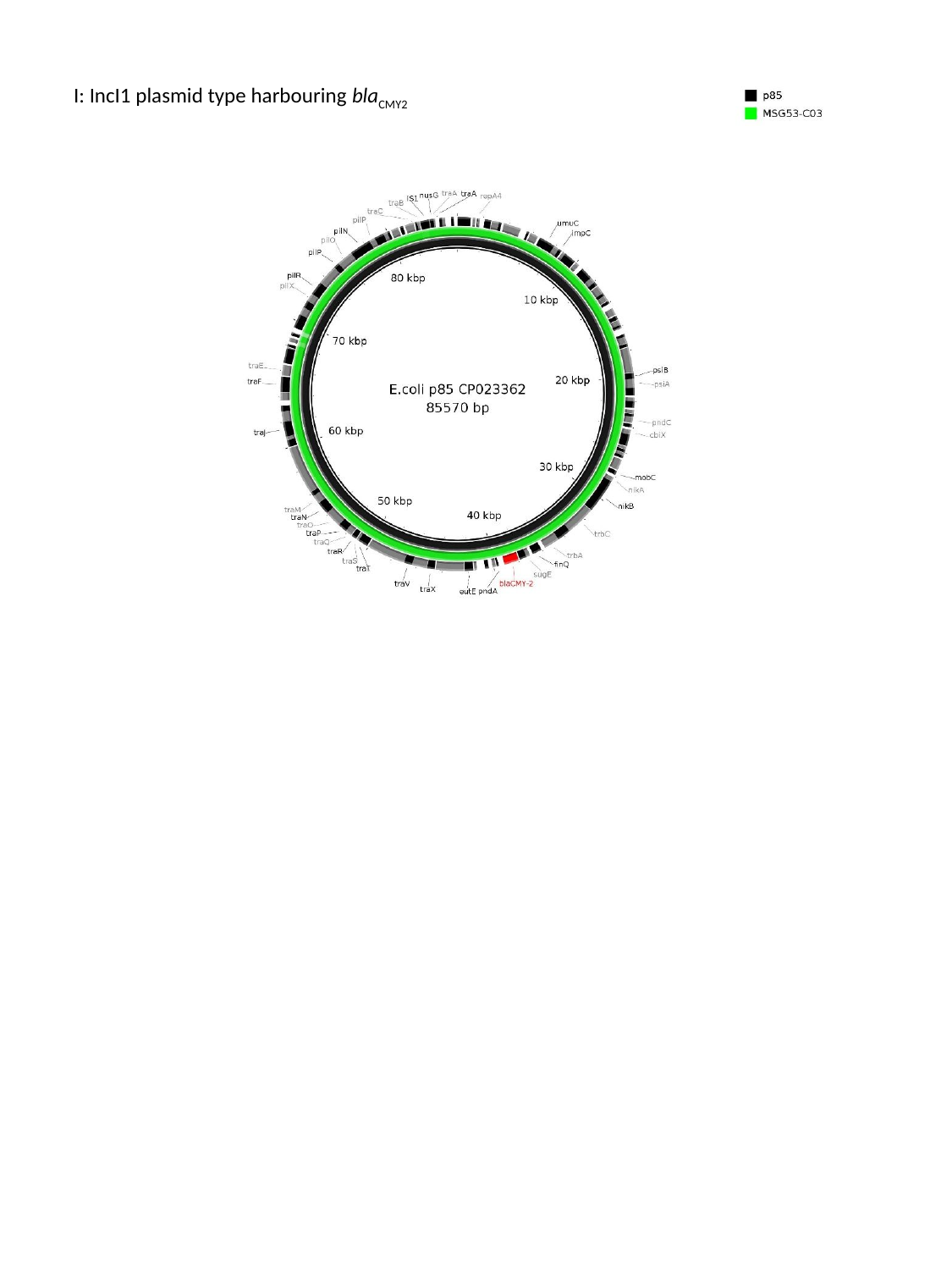

I: IncI1 plasmid type harbouring blaCMY2

## Slide 7
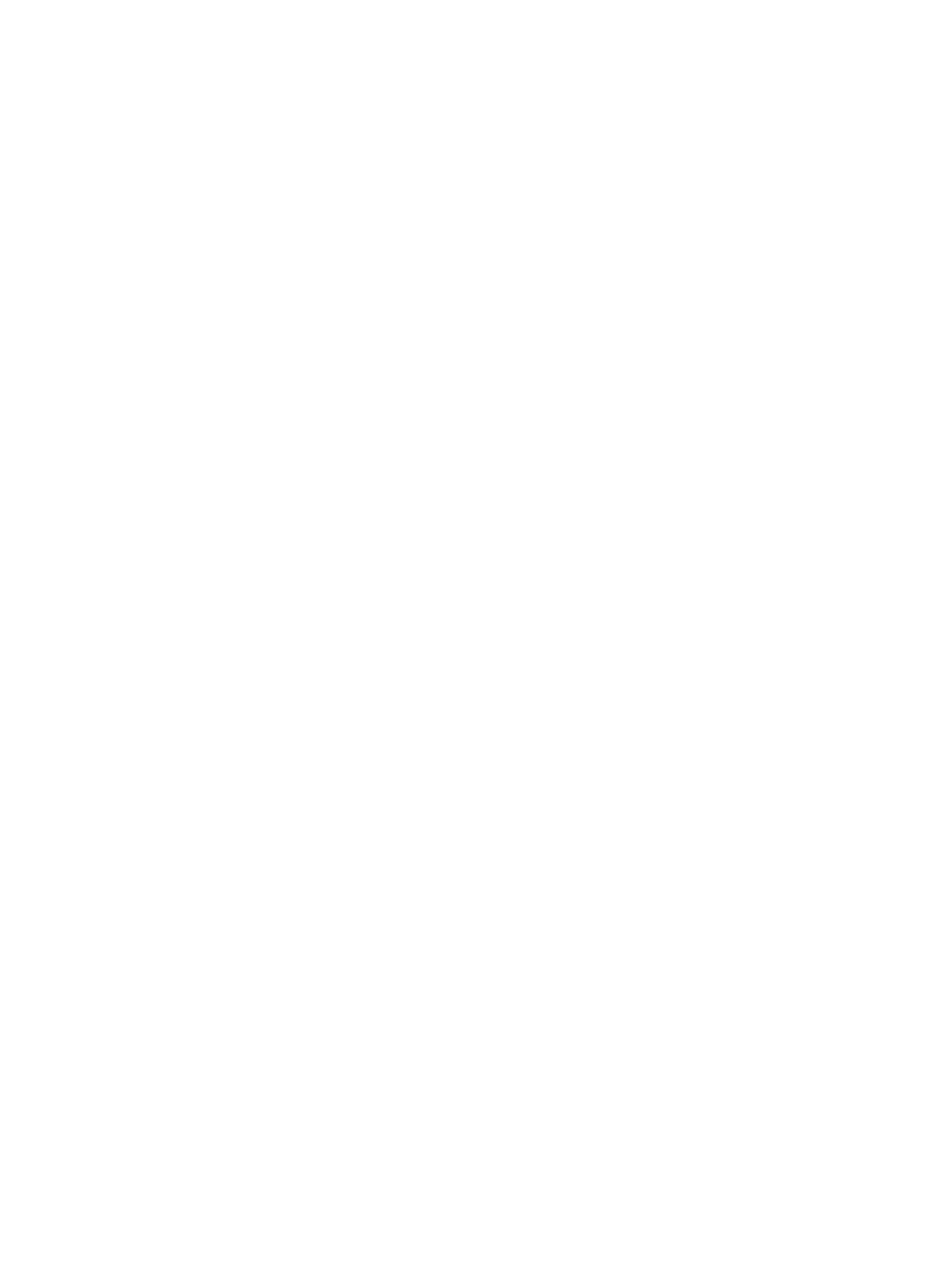

Supplement: Supplementary file 1 [file Presentation_1.pptx]
