# Supplementary material for: Characterizing Antimicrobial Resistant Escherichia coli and Associated Risk Factors in a Cross-Sectional Study of Pig Farms in Great Britain
Source: Front Microbiol. 2020 May 25;11:861. doi: 10.3389/fmicb.2020.00861 (PMC7261845; doi:10.3389/fmicb.2020.00861)
Supplement: Supplementary file 2 [file Table_1.DOC]

**Supplementary Table S1** – Number (N) and proportion (%) of farms recruited for this study, by region and production type

|  | **Farrow-to Finish** | | **Finisher** | | **Total** | |
| --- | --- | --- | --- | --- | --- | --- |
| **Region** | **N** | **%** | **N** | **%** | **N** | **%** |
| **North East** | 3 | 5.4 | 1 | 1.8 | 4 | 7.1 |
| **Yorkshire & the Humber** | 10 | 17.9 | 2 | 3.6 | 12 | 21.4 |
| **Midlands** | 11 | 19.6 | 0 | - | 11 | 19.6 |
| **East of England** | 4 | 7.1 | 3 | 5.4 | 7 | 12.5 |
| **South East** | 4 | 7.1 | 3 | 5.4 | 7 | 12.5 |
| **South West** | 6 | 10.7 | 9 | 16.1 | 15 | 26.8 |
| **Total** | 38 | 67.9 | 18 | 32.1 | 56 | 100.0 |
