# Supplementary material for: Characterizing Antimicrobial Resistant Escherichia coli and Associated Risk Factors in a Cross-Sectional Study of Pig Farms in Great Britain
Source: Front Microbiol. 2020 May 25;11:861. doi: 10.3389/fmicb.2020.00861 (PMC7261845; doi:10.3389/fmicb.2020.00861)
Supplement: Supplementary file 3 [file Table_2.DOCX]

**Supplementary Table S2** – Summary of AMR genes identified in the 492 *E. coli* which are associated with the antimicrobial agents tested in the EFSA panel. Genes which are part of these antimicrobial classes but were not identified in the *E. coli* panel, have not been included. Also, genes conferring resistance to tigecycline, colisitin, or carbapenams, which are included in the EFSA panel, were not identified in the *E. coli* screened and are not given.

| **Antimicrobial class** | **EFSA Antimicrobial** | ***AMR genes*** |
| --- | --- | --- |
| **Clinically Relevant Aminoglycosides** | Gentamicin | *aac(3)-IVa* |
|  |  | *aac(6’)-Iid* |
|  |  | *ant(2”)-Ia* |
|  |  | *aac(3)-IId* |
| ESC | Cefotaxime  Ceftazidime | *bla*_CMY-2_ |
|  |  | *bla*_CTX-M-1_ |
|  |  | *bla*_CTX-M-15_ |
|  |  | *bla*_SHV-12_ |
| **β-lactamase** | Ampicillin | *bla*_TEM-1_ |
|  |  | *bla*_TEM-135_ |
|  |  | *bla*_TEM-30_ |
|  |  | *bla*_TEM-1b_ |
|  |  | *bla*_TEM-1c_ |
|  |  | *bla*_TEM-1d_ |
| **Chloramphenicol/**  **florfenicol** | Chloramphenicol | *catA1* |
|  |  | *catA6* |
|  |  | *cml* |
|  |  | *floR* |
| **Macrolide (Azithromycin)** | Azithromycin | *ermB* |
|  |  | *mefB* |
|  |  | *mphA* |
|  |  | *mphB* |
| **Fluoroquinolone** | Ciprofloxacin | *qnrB19* |
|  |  | *qnrB2* |
|  |  | *qnrS1* |
|  |  | *gyrA^a^* |
|  |  | *parC^a^* |
| **Tetracycline** | Tetracycline | *tet(A)* |
|  |  | *tet(C)* |
|  |  | *tet(D)* |
|  |  | *tet(M)* |
|  |  | *tetA(B)* |
| **Sulphonamide** | Sulfamethoxazole | *sul1* |
|  |  | *sul2* |
| **Trimethoprim** | Trimethoprim | *dfrA1* |
|  |  | *dfrA12* |
|  |  | *dfrA14* |
|  |  | *dfrA15* |
|  |  | *dfrA17* |
|  |  | *dfrA21* |
|  |  | *dfrA25* |
|  |  | *dfrA5* |
|  |  | *dfrA7* |
|  |  | *dfrA8* |
