# Supplementary material for: Characterizing Antimicrobial Resistant Escherichia coli and Associated Risk Factors in a Cross-Sectional Study of Pig Farms in Great Britain
Source: Front Microbiol. 2020 May 25;11:861. doi: 10.3389/fmicb.2020.00861 (PMC7261845; doi:10.3389/fmicb.2020.00861)
Supplement: Supplementary file 4 [file Table_3.DOC]

**Supplementary Table S3 -** Summary of multidrug resistance (MDR) profiles. The number of isolates of an AMR profile is given with the number of farms these isolates were collected from given in parenthesis.

ESBL = Extended spectrum β-lactamase, Amp = Ampicillin, Tet = Tetracycline, Gen = Gentamicin (clinically relevant aminoglycoside), Azm = Azithromycin, Chl = Chloramphenicol, Sul = Sulphonamide, Tmp = Trimethoprim, Fqn = Fluoroquinolone (QRDR and/or PMQR)

| **No of AMR Classes** | AMR profiles | **No of**  **Isolates**  **(farm)** | **% Isolates** | | **% Farms** | |
| --- | --- | --- | --- | --- | --- | --- |
|  |  |  | **Farrow-to-Finish** | **Finisher** | **Farrow-to-Finish** | **Finisher** |
| 8 | Amp,Tet,Gen,Azm,Chl,Sul,Tmp,Fqn | 4 (2) | 1.1 | 0.0 | 5.3 | 0.0 |
| 7 | Amp,Tet,Azm,Chl,Sul,Tmp,Fqn | 12 (8) | 2.6 | 1.9 | 15.8 | 11.1 |
| 7 | Amp,Tet,Gen,Chl,Sul,Tmp,Fqn | 11 (5) | 1.1 | 4.5 | 5.3 | 16.7 |
| 7 | ESBL,Amp,Tet,Azm,Sul,Tmp,Fqn | 1 (1) | 0.3 | 0.0 | 2.6 | 0.0 |
| 7 | Tet,Gen,Azm,Chl,Sul,Tmp,Fqn | 1 (1) | 0.3 | 0.0 | 2.6 | 0.0 |
| 6 | Amp,Tet,Chl,Sul,Tmp,Fqn | 21 (12) | 4.0 | 4.5 | 15.8 | 33.3 |
| 6 | Amp,Tet,Azm,Chl,Tmp,Fqn | 9 (4) | 2.3 | 0.6 | 7.9 | 5.6 |
| 6 | Amp,Tet,Gen,Sul,Tmp,Fqn | 8 (4) | 1.1 | 2.6 | 5.3 | 11.1 |
| 6 | Amp,Tet,Gen,Chl,Sul,Tmp, | 7 (3) | 1.7 | 0.6 | 5.3 | 5.6 |
| 6 | ESBL,Amp,Tet,Sul,Tmp,Fqn | 4 (2) | 1.1 | 0.0 | 5.3 | 0.0 |
| 6 | Amp,Tet,Azm,Chl,Sul,Fqn | 3 (1) | 0.0 | 1.9 | 0.0 | 5.6 |
| 6 | ESBL,Amp,Tet,Chl,Sul,Fqn | 2 (1) | 0.6 | 0.0 | 2.6 | 0.0 |
| 6 | ESBL,Tet,Chl,Sul,Tmp,Fqn | 2 (1) | 0.6 | 0.0 | 2.6 | 0.0 |
| 6 | Amp,Tet,Azm,Chl,Sul,Tmp | 2 (2) | 0.3 | 0.6 | 2.6 | 5.6 |
| 6 | Tet,Azm,Chl,Sul,Tmp,Fqn | 2 (1) | 0.6 | 0.0 | 2.6 | 0.0 |
| 6 | Amp,Gen,Chl,Sul,Tmp,Fqn | 1 (1) | 0.0 | 0.6 | 0.0 | 5.6 |
| 6 | Amp,Tet,Azm,Sul,Tmp,Fqn | 1 (1) | 0.3 | 0.0 | 2.6 | 0.0 |
| 5 | Amp,Tet,Sul,Tmp,Fqn | 23 (12) | 4.0 | 5.8 | 21.1 | 22.2 |
| 5 | Amp,Tet,Chl,Tmp,Fqn | 18 (11) | 2.3 | 6.5 | 15.8 | 27.8 |
| 5 | Amp,Tet,Chl,Sul,Tmp | 9 (5) | 0.0 | 5.8 | 0.0 | 27.8 |
| 5 | Amp,Tet,Gen,Chl,Tmp | 4 (3) | 0.0 | 2.6 | 0.0 | 16.7 |
| 5 | Amp,Tet,Gen,Sul,Fqn | 3 (1) | 0.0 | 1.9 | 0.0 | 5.6 |
| 5 | Tet,Chl,Sul,Tmp,Fqn | 3 (2) | 0.9 | 0.0 | 5.3 | 0.0 |
| 5 | ESBL,Amp,Tet,Sul,Tmp | 2 (1) | 0.0 | 1.3 | 0.0 | 5.6 |
| 5 | ESBL,Tet,Sul,Tmp,Fqn | 2 (1) | 0.6 | 0.0 | 2.6 | 0.0 |
| 5 | Amp,Tet,Azm,Chl,Tmp | 2 (2) | 0.3 | 0.6 | 2.6 | 5.6 |
| 5 | Amp,Tet,Azm,Sul,Tmp | 2 (2) | 0.3 | 0.6 | 2.6 | 5.6 |
| 5 | Amp,Tet,Chl,Sul,Fqn | 2 (1) | 0.6 | 0.0 | 2.6 | 0.0 |
| 5 | ESBL,Amp,Tet,Sul,Fqn | 1 (1) | 0.3 | 0.0 | 2.6 | 0.0 |
| 5 | Amp,Tet,Gen,Sul,Tmp | 1 (1) | 0.3 | 0.0 | 2.6 | 0.0 |
| 4 | Amp,Tet,Chl,Tmp | 12 (9) | 1.7 | 3.9 | 10.5 | 27.8 |
| 4 | Amp,Tet,Sul,Tmp | 11 (8) | 1.4 | 3.9 | 13.2 | 16.7 |
| 4 | Amp,Sul,Tmp,Fqn | 8 (4) | 2.0 | 0.6 | 7.9 | 5.6 |
| 4 | Amp,Tet,Tmp,Fqn | 8 (3) | 1.4 | 1.9 | 5.3 | 5.6 |
| 4 | Tet,Chl,Tmp,Fqn | 7 (3) | 2.0 | 0.0 | 7.9 | 0.0 |
| 4 | Tet,Sul,Tmp,Fqn | 7 (6) | 0.9 | 2.6 | 7.9 | 16.7 |
| 4 | Gen,Sul,Tmp,Fqn | 4 (1) | 1.1 | 0.0 | 2.6 | 0.0 |
| 4 | ESBL,Amp,Tet,Sul | 2 (2) | 0.3 | 0.6 | 2.6 | 5.6 |
| 4 | Amp,Tet,Chl,Sul, | 2 (1) | 0.0 | 1.3 | 0.0 | 5.6 |
| 4 | ESBL,Sul,Tmp,Fqn | 1 (1) | 0.3 | 0.0 | 2.6 | 0.0 |
| 4 | ESBL,Tet,Sul,Fqn | 1 (1) | 0.3 | 0.0 | 2.6 | 0.0 |
| 4 | ESBL,Tet,Sul,Tmp | 1 (1) | 0.3 | 0.0 | 2.6 | 0.0 |
| 4 | Amp,Chl,Tmp,Fqn | 1 (1) | 0.3 | 0.0 | 2.6 | 0.0 |
| 4 | Amp,Tet,Chl,Fqn | 1 (1) | 0.3 | 0.0 | 2.6 | 0.0 |
| 4 | Tet,Azm,Chl,Fqn | 1 (1) | 0.3 | 0.0 | 2.6 | 0.0 |
| 4 | Tet,Azm,Chl,Tmp | 1 (1) | 0.0 | 0.6 | 0.0 | 5.6 |
| 3 | Amp,Tet,Fqn | 17 (6) | 4.0 | 1.9 | 10.5 | 11.1 |
| 3 | Tet,Chl,Tmp | 8 (7) | 1.7 | 1.3 | 13.2 | 11.1 |
| 3 | Tet,Sul,Tmp | 8 (7) | 1.7 | 1.3 | 13.2 | 11.1 |
| 3 | Amp,Tet,Chl | 7 (4) | 1.1 | 1.9 | 5.3 | 11.1 |
| 3 | Amp,Tet,Tmp | 5 (3) | 0.6 | 1.9 | 2.6 | 11.1 |
| 3 | Tet,Sul,Fqn | 3 (2) | 0.9 | 0.0 | 5.3 | 0.0 |
| 3 | Tet,Tmp,Fqn | 3 (3) | 0.6 | 0.6 | 5.3 | 5.6 |
| 3 | Chl,Sul,Fqn | 2 (1) | 0.6 | 0.0 | 2.6 | 0.0 |
| 3 | Tet,Azm,Chl | 2 (2) | 0.3 | 0.6 | 2.6 | 5.6 |
| 3 | Tet,Chl,Sul | 2 (2) | 0.0 | 1.3 | 0.0 | 11.1 |
| 3 | Azm,Chl,Tmp | 1 (1) | 0.3 | 0.0 | 2.6 | 0.0 |
| 3 | ESBL,Sul,Tmp | 1 (1) | 0.0 | 0.6 | 0.0 | 5.6 |
| 3 | ESBL,Tet,Gen | 1 (1) | 0.3 | 0.0 | 2.6 | 0.0 |
| 3 | ESBL,Tet,Fqn | 1 (1) | 0.3 | 0.0 | 2.6 | 0.0 |
| 3 | ESBL,Tet,Sul | 1 (1) | 0.3 | 0.0 | 2.6 | 0.0 |
| 3 | Amp,Chl,Tmp | 1 (1) | 0.3 | 0.0 | 2.6 | 0.0 |
| 3 | Amp,Sul,Fqn | 1 (1) | 0.0 | 0.6 | 0.0 | 5.6 |
| 3 | Tet,Chl,Fqn | 1 (1) | 0.0 | 0.6 | 0.0 | 5.6 |
| 2 | Tet,Fqn | 16 (8) | 4.6 | 0.0 | 21.1 | 0.0 |
| 2 | Amp,Tet | 14 (10) | 2.9 | 2.6 | 15.8 | 22.2 |
| 2 | Tet,Tmp | 13 (8) | 2.6 | 2.6 | 10.5 | 22.2 |
| 2 | Tet,Sul | 11 (6) | 2.6 | 1.3 | 10.5 | 11.1 |
| 2 | Tet,Chl | 9 (6) | 1.7 | 1.9 | 10.5 | 11.1 |
| 2 | ESBL,Tet | 4 (1) | 1.1 | 0.0 | 2.6 | 0.0 |
| 2 | Sul,Tmp | 3 (3) | 0.9 | 0.0 | 7.9 | 0.0 |
| 2 | Azm,Chl | 1 (1) | 0.3 | 0.0 | 2.6 | 0.0 |
| 2 | Chl,Fqn | 1 (1) | 0.3 | 0.0 | 2.6 | 0.0 |
| 2 | Chl,Tmp | 1 (1) | 0.3 | 0.0 | 2.6 | 0.0 |
| 2 | Amp,Gen | 1 (1) | 0.0 | 0.6 | 0.0 | 5.6 |
| 2 | Amp,Fqn | 1 (1) | 0.3 | 0.0 | 2.6 | 0.0 |
| 1 | Tet | 32 (18) | 4.6 | 10.3 | 26.3 | 44.4 |
| 1 | Fqn | 5 (3) | 1.1 | 0.6 | 5.3 | 5.6 |
| 1 | Amp | 2 (2) | 0.6 | 0.0 | 5.3 | 0.0 |
| 1 | Sul | 2 (2) | 0.6 | 0.0 | 5.3 | 0.0 |
| 1 | ESBL | 1 (1) | 0.3 | 0.0 | 2.6 | 0.0 |
| 1 | Tmp | 1 (1) | 0.3 | 0.0 | 2.6 | 0.0 |
| 0 |  | 78 (30) | 19.3 | 7.1 | 63.2 | 33.3 |
