# Supplementary material for: Characterizing Antimicrobial Resistant Escherichia coli and Associated Risk Factors in a Cross-Sectional Study of Pig Farms in Great Britain
Source: Front Microbiol. 2020 May 25;11:861. doi: 10.3389/fmicb.2020.00861 (PMC7261845; doi:10.3389/fmicb.2020.00861)
Supplement: Supplementary file 5 [file Table_4.DOC]

**Supplementary Table S4** – Summary of plasmid Inc-types and AMR gene combinations present in isolates.

| **No. of**  **isolates** | **Plasmid / AMR gene combinations** |
| --- | --- |
| 48 | IncQ1 + *strA, strB, sul2* |
| 19 | IncI1a + *qnrS1* |
| 9 | IncFIC, IncFII(pSFO) + *strA, strB, bla*_TEM-1b_*, int1, sul2, tet(A), dfrA14* |
| 6 | IncFIC IncFII(pSFO) + *bla*_TEM-135_*, qnrS1, tet(A)* |
| 5 | IncQ1 + *strA, strB, floR, sul2, tet(A)* |
| 5 | IncX3 + *qnrS1* |
| 4 | IncFIB(AP001918) + *strA, strB* |
| 4 | IncFIC, IncFII(pSFO) + *tet(A)* |
| 4 | IncFII + *tet(A)* |
| 4 | IncHI2A + *strA*, *strB*, *bla*_CMY-2_ |
| 4 | IncI1a + *ant(3”)-Ia*, *strA*, *strB*, int1, *tet(A),* *dfrA1* |
| 4 | p0111 + *aadA2*, *ant(3)-Ia*, *cmlA1*, int1 |
| 3 | ColRNAI + *strA*, *strB*, *sul2* |
| 3 | ColRNAI + *strA*, *strB*, *sul2*, *dfrA14* |
| 3 | IncFIB(AP001918), IncFIC, IncFII(pSFO) + *strA*, *strB*, *tetA(B)* |
| 3 | IncFIC, IncFII(pSFO) + *strA*, *strB* |
| 3 | IncFIC, IncFII(pSFO) + *bla*_TEM-1b_ |
| 3 | IncFII(pCoo) + *aac(3)-IId*, *bla*_TEM-1b_ |
| 3 | IncI1a + *ant(3”)-Ia*, int1, *sul3*, *tet(A)* |
| 3 | IncI1a + *tet(A)* |
| 3 | IncQ1 + *strA*, int1, *sul2* |
| 3 | IncX1, IncX3 + *bla*_TEM-1b_, *qnrS1* |
| 2 | ColRNAI + *strA*, *sul2*, *dfrA14* |
| 2 | ColRNAI + *qnrB5* |
| 2 | IncB/O/K/Z, IncQ1 + *strA*, *strB*, *sul2*, *dfrA14* |
| 2 | IncFIA, IncFIC, IncFII(pSFO) + *strA*, *strB*, *bla*_TEM-1b_, *sul2* |
| 2 | IncFIA, IncFIC, IncFII(pSFO) + *bla*_TEM-1b_, *tet(A)* |
| 2 | IncFIB(AP001918), IncFIC, IncFII(pSFO) + *tet(A)* |
| 2 | IncFIC, IncA/C2, IncR + *strA*, *strB*, *tet(A)* |
| 2 | IncFIC, IncFII(pSFO) + bla_TEM-1b_, *tet(A),* *dfrA8* |
| 2 | IncI1a + *sul2*, *tet(A)* |
| 2 | IncQ1 + *ant3-Ia*, *strA*, *strB*, int1, *mphB*, *sul2*, *sul3*, *dfrA1* |
| 2 | IncQ1 + *strA*, *strB*, int1, *sul2* |
| 2 | IncQ1 + *sul2* |
| 2 | IncR + *strA*, *strB*, *tetA(B)* |
| 2 | IncX1 + *bla*_TEM-1b_ |
| 2 | IncX1, IncX3 + *aadA2*, *ant(3”)-Ia*, *cmlA1*, int1, *mefB*, *dfrA1*2 |
| 1 | Col8282 + *tet(A)* |
| 1 | ColRNAI + *strA*, *strB*, *sul2*, *tet(A)* |
| 1 | ColRNAI + *strA*, *strB*, *sul2*, *tet(A),* *dfrA14* |
| 1 | ColRNAI + *strB* |
| 1 | ColRNAI + *sul2* |
| 1 | IncA/C2 + *bla*_CMY-2_ |
| 1 | IncA/C2 + *bla*_CMY-2_, *sul2* |
| 1 | IncFIA(HI1), IncFIB(Kpn3) + *aadA2*, *ant(3”)-Ia*, *cmlA1*, int1, *tet(A)*, *dfrA1*2 |
| 1 | IncFIA(HI1), IncFIB(Kpn3) + *bla*_TEM-1b_, *qnrS1* |
| 1 | IncFIA, IncFIC, IncFII(pSFO) + *bla*_TEM-1b_ |
| 1 | IncFIB(AP001918) + int1, *tet(A)*, *dfrA5* |
| 1 | IncFIB(AP001918) + *tet(A)* |
| 1 | IncFIB(AP001918), IncFIC(FII), IncFII(pSFO) + *aadA2*, *ant(3”)-Ia*, *cmlA1*, int1, *dfrA1*2 |
| 1 | IncFIB(AP001918), IncFIC(FII), IncFII(pSFO) + int1 |
| 1 | IncFIB(AP001918), IncFIC(FII), IncFII(pSFO) + *tetA(B)* |
| 1 | IncFIB(K) + int2 |
| 1 | IncFIC, IncFII(pSFO) + *aadA2*, *ant(3”)-Ia*, *cmlA1*, int1 |
| 1 | IncFIC, IncFII(pSFO) + *aadA2*, *ant(3”)-Ia*, *cmlA1*, int1, *mefB* |
| 1 | IncFIC, IncFII(pSFO) + *aadA2*, *ant(3”)-Ia*, *cmlA1*, int1, *mefB*, *dfrA1*2 |
| 1 | IncFIC, IncFII(pSFO) + *aadA2*, *ant(3”)-Ia*, *cmlA1*, int1, *tet(A)*, *dfrA1*2 |
| 1 | IncFIC, IncFII(pSFO) + *strA*, *strB*, bla_TEM-1b_, *sul2* |
| 1 | IncFIC, IncFII(pSFO) + *strA*,*sul2*, *dfrA14* |
| 1 | IncFIC, IncFII(pSFO) + *bla*_TEM-1b_, *tet(A)* |
| 1 | IncFIC, IncFII(pSFO) + *mdfA* |
| 1 | IncFIC, IncFII(pSFO), IncQ1 + *ant(3”)-Ia*, *strA*, *strB*, int1, *mphB*, *sul2*, *sul3*, *dfrA1* |
| 1 | IncFII(pCoo) + *tet(A)* |
| 1 | IncFII(pRSB107) + *tetA(B)* |
| 1 | IncFII + *aadA2*, *ant(3”)-Ia*, *cmlA1*, int1, *mefB*, *dfrA1*2 |
| 1 | IncFII + *aadA2*, *ant(3”)-Ia*, *cmlA1*, int1, *tetA(B)* |
| 1 | IncFII + *strA*, *strB*, *sul2* |
| 1 | IncFII + *strA*, *strB*, *sul2*, *dfrA14* |
| 1 | IncFII + int1, *tet(A)*, *dfrA5* |
| 1 | IncFII, IncR + *aadA2*, *ant(3”)-Ia*, *strA*, *strB*, *cmlA1*, int1, *tetA(B)* |
| 1 | IncI1a + *aadA13*, int1, *qnrS1* |
| 1 | IncI1a + *aadA2*, *ant(3”)-Ia*, *cmlA1*, int1, *mefB* |
| 1 | IncI1a + *aadA5*, int1, *sul2*, *dfrA1*7 |
| 1 | IncI1a + *aadA5*, int1, *tet(A)*, *dfrA1*7 |
| 1 | IncI1a + *bla*_CMY-2_ |
| 1 | IncI1a + *bla*_TEM-1b_, int1, *sul2*, *tet(A)*, *dfrA1* |
| 1 | IncI1a + int1, *dfrA1* |
| 1 | IncI1a + *sul3*, *tet(A)* |
| 1 | IncI1a, p0111 + *bla*_CTX-M-1_, *sul2* |
| 1 | IncN + *strA*, *strB*, *sul2*, *tet(A)*, *dfrA14* |
| 1 | IncN + *qnrB20*, *sul3*, *tet(A)*, *dfrA25* |
| 1 | IncQ1 + *ant(3”)-Ia*, *strA*, *strB*, *bla*_TEM-1b_, int1, *mphB*, *sul2*, *sul3*, *tet(A)*, *dfrA1* |
| 1 | IncQ1 + *ant(3”)-Ia*, *strA*, *strB*, int1, *sul2*, *sul3*, *dfrA1* |
| 1 | IncQ1 + *strA*, *strB*, *sul2*, *tet(A)*, *dfrA14* |
| 1 | IncQ1 + *floR*, *sul2* |
| 1 | IncR + *ant(3”)-Ia*, *cmlA1*, int1 |
| 1 | IncR + *strA*, *strB* |
| 1 | IncR + *bla*_TEM-1b_ |
| 1 | IncX2 + *bla*_TEM-1b_, *qnrB5*, *tet(A)* |
| 1 | IncX2 + *tet(A)* |
