# Supplementary material for: Characterizing Antimicrobial Resistant Escherichia coli and Associated Risk Factors in a Cross-Sectional Study of Pig Farms in Great Britain
Source: Front Microbiol. 2020 May 25;11:861. doi: 10.3389/fmicb.2020.00861 (PMC7261845; doi:10.3389/fmicb.2020.00861)
Supplement: Supplementary file 6 [file Table_5.doc]

**Supplementary Table S5** – Summary of GyrA and ParC QRDR substitutions found in *E.coli* from different isolation plates.

| **GyrA SNP ^a^** | | | | | | |
| --- | --- | --- | --- | --- | --- | --- |
| **GyrA** | **83/87^a^** | **1mg/L**  **cefotaxime** | **1mg/L**  **ciprofloxacin** | **No Antibiotic** | **CRE** | **Total** |
|  | S / D (WT) | 30 | 58 | 252 | 4 | 333 |
|  | L / D | 1 | 51 | 6 | 0 | 58 |
|  | L / N | 6 | 69 | 5 | 0 | 80 |
|  | S / A | 0 | 0 | 1 | 0 | 1 |
|  | S / N | 0 | 1 | 0 | 0 | 1 |
|  | S / Y | 0 | 10 | 1 | 0 | 11 |
|  | L / E | 0 | 8 | 0 | 0 | 8 |
| **ParC SNP** | | | | | | |
| **ParC** | **80 / 84 ^b^** | **1mg/L**  **cefotaxime** | **1mg/L**  **ciprofloxacin** | **No Antibiotic** | **CRE** | **Total** |
|  | S / E (WT) | 29 | 125 | 253 | 3 | 410 |
|  | I / E | 7 | 71 | 1 | 1 | 80 |
|  | R / E | 0 | 1 | 0 | 0 | 1 |
|  | R / K | 1 | 0 | 0 | 0 | 1 |
| **Double-Serine mutation ^c^** | | | | | | |
|  | | **1mg/L**  **cefotaxime** | **1mg/L**  **ciprofloxacin** | **No Antibiotic** | **CRE** | **Total** |
| **GyrA S83 & ParC S80** | | 5 | 68 | 0 | 0 | 73 |

^a^ SNPs at position 83 and 87 are located within the quinolone resistance determining regions (QRDR) in GyrA. ^b^ SNPs at position 80 and 84 are located within the QRDR in ParC. ^c^ Double-serine mutation at gryA S83 and ParC S80 as described by Fuzi et al., 2017
