# Supplementary material for: Characterizing Antimicrobial Resistant Escherichia coli and Associated Risk Factors in a Cross-Sectional Study of Pig Farms in Great Britain
Source: Front Microbiol. 2020 May 25;11:861. doi: 10.3389/fmicb.2020.00861 (PMC7261845; doi:10.3389/fmicb.2020.00861)
Supplement: Supplementary file 7 [file Table_6.doc]

**Supplementary Table S6**. *E.coli* isolates recovered from *Brilliance*^TM^ carbapenem-resistant Enterobacteriaceae (CRE) plates and the carbapenam susceptibilities and AMR gene content from WGS. ^a^ The EUCAST ECOFF values are 0.125 mg/L Meropenem, 0.5 mg/L Imipenem and 0.125 mg/L for Doripenem. **^b^** Gene presence determined from WGS using APHA SeqFinder. ^6^

|  | **Carbapenam MIC^a^** | | | **AMR genotype^b^** | | | | | |
| --- | --- | --- | --- | --- | --- | --- | --- | --- | --- |
| **Isolate ID** | **Meropenem** | **Imipenem** | **Doripenem** | **Beta-lactam** | **Streptomycin** | **Trimethoprim sulfamethoxazole** | **Tetracycline** | **Quinolone** | **Macrolide** |
| MSG17-C19 | 0.03 | 0.06 | 0.03 | *bla*_CTX-M1_, *bla*_TEM-1B_ | *strAB* | *dfrA5, sul2* | *tet(B)* | *-* | *mphA* |
| MSG17-C20 | 0.015 | 0.06 | 0.03 | *bla*_CTX-M15_, *bla*_TEM-1B_ | *strAB* | *dfrA14, sul2* | *tet(A)* | *qnrS1* | *-* |
| MSG25-C19 | 0.03 | 0.125 | 0.06 | *bla*_CTX-M15_, *bla*_TEM-1B_ | *strAB* | *dfrA14*, *sul2* | *tet(A)* | *qnrS1* | *-* |
| MSG25-C20 | 0.03 | 0.125 | 0.06 | *bla*_CTX-M15_, *bla*_TEM-1B_ | *strAB* | *dfrA14*, *sul2* | *tet(A)* | *qnrS1* | *-* |
