# Supplementary material for: Characterizing Antimicrobial Resistant Escherichia coli and Associated Risk Factors in a Cross-Sectional Study of Pig Farms in Great Britain
Source: Front Microbiol. 2020 May 25;11:861. doi: 10.3389/fmicb.2020.00861 (PMC7261845; doi:10.3389/fmicb.2020.00861)
Supplement: Supplementary file 8 [file Table_7.DOCX]

**Supplementary Table S7** - List of *E. coli* MLST sequence types recovered from 56 pig farms, and the number of MDR isolates. There are an additional 17 new STs, which were not in the PubMLST database and have not been listed below.

| **MLST Sequence type** | Clonal complex | **Pathotype^a^** | **Number of isolates** | **Number of farms** | MDR | **Percentage of human isolates from the total number (in brackets) in PubMLST database ^b^** |
| --- | --- | --- | --- | --- | --- | --- |
| 10 | ST10 Cplx | ExPEC | 46 | 26 | 23 | 17.2% (6608) |
| 48 |  |  | 13 | 7 | 12 | 11.7% (623) |
| 617 |  |  | 3 | 1 | 3 | 42.4% (262) |
| 34 |  |  | 2 | 2 | 1 | 38.3% (303) |
| 167 |  |  | 1 | 1 | 1 | 41.5% (383) |
| 209 |  |  | 1 | 1 | 1 | 33.3% (6) |
| 218 |  |  | 1 | 1 | 0 | 17.1% (76) |
| 227 |  |  | 1 | 1 | 1 | 53.1% (49) |
| 101 | ST101 Cplx |  | 30 | 12 | 24 | 18.2% (716) |
| 131 | ST131 Cplx | ExPEC | 10 | 4 | 7 | 52.3% (7484) |
| 58 | ST155 Cplx |  | 55 | 21 | 46 | 14.0% (1190) |
| 155 |  |  | 1 | 1 | 0 | 10.0% (909) |
| 156 | ST156 Cplx |  | 2 | 1 | 1 | 21.0% (257) |
| 93 | ST168 Cplx |  | 3 | 2 | 2 | 4.2% (1799) |
| 20 | ST20 Cplx |  | 10 | 5 | 6 | 14.8% (162) |
| 206 | ST206 Cplx |  | 1 | 1 | 0 | 14.4% (215) |
| 88 | ST23 Cplx |  | 25 | 11 | 9 | 17.9% (862) |
| 23 |  |  | 8 | 6 | 7 | 14.6% (560) |
| 367 |  |  | 1 | 1 | 0 | 9.5% (21) |
| 410 |  |  | 1 | 1 | 1 | 34.8% (799) |
| 316 | ST278 Cplx |  | 1 | 1 | 1 | 21.5% (65) |
| 57 | ST350 Cplx |  | 3 | 1 | 0 | 13.8% (159) |
| 38 | ST38 Cplx | ExPEC | 2 | 1 | 2 | 45.3% (1339) |
| 398 | ST398 Cplx |  | 1 | 1 | 1 | 15.4% (169) |
| 46 | ST46 Cplx |  | 1 | 1 | 0 | 29.3% (191) |
| 162 | ST469 Cplx |  | 25 | 9 | 24 | 16.8% (537) |
| 648 | ST648 Cplx | Bacteraemia | 1 | 1 | 1 | 41.1% (828) |
| 69 | ST69 Cplx | ExPEC | 4 | 3 | 3 | 38.9% (1691) |
| 73 | ST73 Cplx | ExPEC | 1 | 1 | 0 | 35.8% (1812) |
| 641 | ST86 Cplx |  | 16 | 9 | 8 | 9.3% (311) |
| 453 |  |  | 2 | 2 | 0 | 36.3% (182) |
| 86 |  |  | 1 | 1 | 0 | 8.7% (46) |
| 744 |  |  | 37 | 13 | 35 | 33.9% (313) |
| 117 |  | ExPEC | 25 | 12 | 18 | 9.4% (1091) |
| 542 |  |  | 11 | 8 | 2 | 4.0% (125) |
| 359 |  |  | 8 | 4 | 8 | 32.8% (58) |
| 3630 |  |  | 6 | 3 | 0 | 0.0% (3) |
| 154 |  |  | 5 | 5 | 2 | 17.8% (213) |
| 4175 |  |  | 5 | 5 | 5 | 0.0% (3) |
| 6096 |  |  | 5 | 2 | 1 | 0.0% (11) |
| 540 |  |  | 4 | 4 | 2 | 7.6% (236) |
| 767 |  |  | 4 | 2 | 2 | 10.7% (28) |
| 1114 |  |  | 4 | 2 | 2 | 5.6% (36) |
| 75 |  |  | 3 | 3 | 2 | 6.3% (64) |
| 953 |  |  | 3 | 1 | 0 | 0.0% (4) |
| 993 |  |  | 3 | 2 | 2 | 15.8% (38) |
| 1101 |  |  | 3 | 2 | 0 | 0.0% (13) |
| 4429 |  |  | 3 | 1 | 0 | 18.2% (11) |
| 6438 |  |  | 3 | 2 | 1 | 12.5% (8) |
| 710 |  |  | 2 | 2 | 0 | 11.1% (18) |
| 847 |  |  | 2 | 2 | 1 | 12.2% (74) |
| 877 |  |  | 2 | 2 | 2 | 8.7% (23) |
| 1112 |  |  | 2 | 2 | 1 | 0.0% (30) |
| 1431 |  |  | 2 | 2 | 2 | 20.3% (64) |
| 2436 |  |  | 2 | 1 | 0 | 12.5% (8) |
| 2496 |  |  | 2 | 2 | 2 | 8.3% (12) |
| 2521 |  |  | 2 | 1 | 0 | 6.6% (61) |
| 3595 |  |  | 2 | 1 | 0 | 0.0% (5) |
| 4247 |  |  | 2 | 1 | 0 | 0.0% (4) |
| 4379 |  |  | 2 | 1 | 0 | 0.0% (2) |
| 5771 |  |  | 2 | 2 | 1 | 100.0% (1) |
| 1 |  |  | 1 | 1 | 1 | 9.4% (32) |
| 42 |  |  | 1 | 1 | 1 | 0.0% (18) |
| 224 |  |  | 1 | 1 | 1 | 21.8% (243) |
| 345 |  |  | 1 | 1 | 0 | 15.1% (152) |
| 442 |  |  | 1 | 1 | 0 | 67.1% (516) |
| 683 |  |  | 1 | 1 | 0 | 7.9% (38) |
| 717 |  |  | 1 | 1 | 1 | 16.7% (6) |
| 846 |  |  | 1 | 1 | 1 | 44.4% (9) |
| 871 |  |  | 1 | 1 | 0 | 32.0% (25) |
| 910 |  |  | 1 | 1 | 0 | 0.0% (6) |
| 925 |  |  | 1 | 1 | 1 | 19.0% (21) |
| 939 |  |  | 1 | 1 | 1 | 13.8% (29) |
| 1056 |  |  | 1 | 1 | 1 | 12.1% (33) |
| 1068 |  |  | 1 | 1 | 0 | 33.3% (3) |
| 1115 |  |  | 1 | 1 | 0 | 0.0% (5) |
| 1202 |  |  | 1 | 1 | 0 | 25.0% (8) |
| 1286 |  |  | 1 | 1 | 1 | 31.1% (61) |
| 1433 |  |  | 1 | 1 | 0 | 42.9% (7) |
| 1494 |  |  | 1 | 1 | 1 | 0.0% (21) |
| 2025 |  |  | 1 | 1 | 0 | 11.5% (26) |
| 2077 |  |  | 1 | 1 | 0 | 0.0% (16) |
| 2325 |  |  | 1 | 1 | 1 | 2.9% (68) |
| 2354 |  |  | 1 | 1 | 0 | 0.0% (16) |
| 2526 |  |  | 1 | 1 | 1 | 28.6% (7) |
| 3205 |  |  | 1 | 1 | 1 | 33.3% (6) |
| 3631 |  |  | 1 | 1 | 1 | 0.0% (3) |
| 4038 |  |  | 1 | 1 | 0 | 11.6% (43) |
| 4541 |  |  | 1 | 1 | 0 | 50.0% (2) |
| 4580 |  |  | 1 | 1 | 0 | 0.0% (6) |
| 4619 |  |  | 1 | 1 | 0 | 0.0% (0) |
| 5236 |  |  | 1 | 1 | 0 | 52.6% (19) |
| 5281 |  |  | 1 | 1 | 0 | 18.2% (11) |
| 5409 |  |  | 1 | 1 | 0 | 0.0% (8) |
| 5752 |  |  | 1 | 1 | 0 | 0.0% (1) |
| 5759 |  |  | 1 | 1 | 0 | 0.0% (6) |
| 5995 |  |  | 1 | 1 | 1 | 0.0% (12) |
| 6198 |  |  | 1 | 1 | 1 | 0.0% (2) |
| 6517 |  |  | 1 | 1 | 0 | 0.0% (1) |
| 6730 |  |  | 1 | 1 | 0 | 0.0% (5) |

^a^ Sequence types commonly associated with Extra-intestinal pathogenic *E. coli* (ExPEC) infections (Gibreel et al., 2012; Manges and Johnson, 2012; Horner et al., 2014; Kallonen et al., 2017; Day et al., 2019).

^b^ The percentage of isolates listed as originating from humans in the PubMLST database. The number in brackets reflects the total number of isolates of a given ST, from any source including human. The PubMLST database was accessed on 20^th^ November 2019.
